# PFOCUS study protocol: a prospective case–control study investigating genetic, haemodynamic, and platelet function in migraine with aura with or without patent foramen ovale

**DOI:** 10.3389/fneur.2026.1952744

**Published:** 2026-10-16

**Authors:** Matteo Paolucci, Giacomo Urbinati, Ludovica Migliaccio, Alice Marucci, Valentina Favoni, Davide Mascarella, Flavia Palombo, Valerio Carelli, Mario Sebastiani, Silvia Stagni, Rita Mancini, Sabina Cevoli, Andrea Zini

**Affiliations:** 1 Department of Neurology and Stroke Centre, Maggiore Hospital, IRCCS Istituto delle Scienze Neurologiche di Bologna, Bologna, Italy; 2 Department of Biomedical and Neuromotor Sciences (DIBINEM), Alma Mater Studiorum–University of Bologna, Bologna, Italy; 3 Programma Cefalee ed Algie Facciali, IRCCS Istituto delle Scienze Neurologiche di Bologna (ISNB), Bologna, Italy; 4 Programma di Neurogenetica, IRCCS Istituto delle Scienze Neurologiche di Bologna (ISNB), Bologna, Italy; 5 Department of Neuroradiology, Maggiore Hospital, IRCCS Istituto delle Scienze Neurologiche di Bologna, Bologna, Italy; 6 Laboratorio Unico Metropolitano (LUM), AUSL Bologna, Bologna, Italy

**Keywords:** cerebrovascular reactivity, dynamic cerebral autoregulation, migraine with aura, Notch3, patent foramen ovale, right-to-left shunt, transcranial Doppler, whole-exome sequencing

## Abstract

**Background:**

Migraine with aura (MA) is associated with an increased risk of ischemic stroke, particularly in young individuals without conventional vascular risk factors. A growing body of evidence indicates a strong association between MA and patent foramen ovale (PFO), the prevalence of which is up to three times higher in patients with MA than in the general population. The mechanisms underlying this association remain incompletely understood and are likely to extend beyond paradoxical embolism.

**Methods and design:**

PFOCUS is a prospective, monocentric, observational case–control study designed to investigate the genetic, neurosonological, and hematological determinants linking MA and PFO. Specifically, the study evaluates whether genetic variants, particularly within the Notch signaling pathway, contribute to the coexistence of PFO and MA, and whether these patients display distinct cerebrovascular and prothrombotic profiles. As exploratory aims, the study will assess whether aura phenotypes and the distribution of MRI lesions are associated with different haemodynamic and prothrombotic profiles, potentially identifying clinically relevant subgroups within MA.

A total of 240 patients with MA will be enrolled and classified as right-to-left shunt (RLS)-positive or RLS-negative on the basis of contrast-enhanced transcranial Doppler (TCD), used as a surrogate marker of PFO. Participants will undergo comprehensive phenotyping including genetic testing by whole-exome sequencing, neurosonological assessment of right-to-left shunt, cerebral microemboli detection, dynamic cerebral autoregulation and cerebral vasomotor reactivity (VMR), and platelet function testing. The primary endpoint is the difference in the frequency of NOTCH3 gene variants between groups. Secondary endpoints include associations with other panel genes, correlation with shunt size, and differences in haemodynamic and platelet function parameters. ClinicalTrials.gov: NCT07349004.

**Discussion:**

PFOCUS aims to identify a biologically distinct phenotype of MA associated with PFO, potentially driven by genetic predisposition and vascular dysfunction. Its findings may inform stroke risk stratification and open new avenues for personalized preventive strategies in patients with MA.

**Clinical trial registration:**

ClinicalTrials.gov, identifier NCT07349004 (https://clinicaltrials.gov/study/NCT07349004).

## Introduction

Migraine with aura (MA) is a common neurological condition affecting up to 5% of the general population and has been consistently associated with an increased risk of ischemic stroke (1–3). Notably, this risk is particularly evident in younger patients who lack traditional vascular risk factors, suggesting the involvement of alternative pathophysiological mechanisms (2, 4).

Among these mechanisms, the presence of a patent foramen ovale (PFO) has emerged as a key factor. While PFO is found in approximately 25% of the general population, its prevalence is two to three times higher in individuals with MA (5, 6). Moreover, in young patients with cryptogenic stroke and frequent aura, PFO prevalence may reach up to 93% (7). These observations strongly suggest a pathophysiological link between PFO and MA. Migraine itself, however, is not a purely vascular disorder: it is currently understood as a complex disorder of nervous system function, involving cortical and thalamic networks, activation of the trigeminovascular system and neuropeptides such as calcitonin gene-related peptide (CGRP) and pituitary adenylate cyclase-activating polypeptide (PACAP) (8). PFOCUS does not address migraine pathogenesis as a whole, but the specific mechanisms linking MA to a right-to-left shunt, for which genetic and haemodynamic determinants are the relevant candidates.

Traditionally, the association between PFO and stroke has been attributed to paradoxical embolism. However, accumulating evidence indicates that additional mechanisms may be involved. *In situ* thrombus formation within the PFO has been demonstrated by cardiac optical coherence tomography in up to 85% of patients with stroke or with MA and PFO, but only in 23% of patients with migraine without aura and never (0%) in healthy individuals with PFO, in association with endocardial abnormalities (9). Furthermore, patients with MA and PFO display a prothrombotic profile that regresses after PFO closure (10). Platelet activation and oxidative stress appear to play central roles in this process, possibly driven by mechanical stress during the passage of blood through the shunt; oxidative stress has also been proposed as a basis for the endothelial dysfunction observed in patients with migraine (11).

The reason why the foramen ovale remains patent in such a large proportion of the population is unclear. After birth, its closure is a process of endothelial-to-mesenchymal transition regulated by Notch signaling; an animal study demonstrated high expression of Notch1 and Notch3 receptors, the ligand Jagged1, and the effector HRT1 in the endocardium of the foramen ovale region during this transition (12). Interestingly, mutations in the NOTCH3 gene underlie cerebral autosomal-dominant arteriopathy with subcortical infarcts and leukoencephalopathy (CADASIL), the most common hereditary cause of ischemic stroke, in which MA is part of the clinical spectrum in 20%−40% of cases, often as a presenting symptom (13).

NOTCH3 (Notch Receptor 3), located on chromosome 19 (19p13.2–13.1), is crucial for vascular development, particularly the maturation of vascular smooth muscle cells (14). NOTCH3 mutations in the general population are more frequent than previously thought and produce a broad spectrum of clinical presentations, ranging from overt CADASIL to CADASIL-like syndromes and even subtler phenotypes (15). NOTCH3 is a highly variable gene with multiple common (≥5%) and rare single nucleotide polymorphisms (SNPs), some of which have been hypothesized to alter the response of vascular smooth muscle cells to hypertension (16). To date, however, neither NOTCH3 nor any other gene has been associated with the presence of PFO (17).

Some NOTCH3 polymorphisms have been associated with migraine (18, 19): the rs3815188 (C381T) polymorphism shows a weak association with migraine, whereas rs1043994 (G684A) is significantly associated with MA, although it is unknown whether carriers were also PFO carriers. In a large elderly population, four common and nine rare SNPs were significantly associated with the presence and progression of white-matter hyperintensities, but only in hypertensive subjects (16), suggesting that NOTCH3 dysfunction may entail an altered vascular response to hypertension. Consistently, impaired dynamic cerebral autoregulation has been reported in migraineurs with large right-to-left shunts (20). With regard to arteriolar tone regulation, which underlies cerebral autoregulation, patients with MA show a peculiar cerebral vasomotor reactivity (VMR) to CO__2__, with most studies reporting increased VMR despite some conflicting data (21, 22). Finally, markers of platelet activation, circulating platelet aggregates, Tissue Factor expression, and thrombin generation are higher in patients with MA and PFO than in healthy controls, indicating a greater prothrombotic potential secondary to platelet hyperactivation—a condition inhibited by clopidogrel but not by aspirin (10).

Taken together, these observations suggest that patients with MA and PFO may represent a distinct biological phenotype characterized by genetic susceptibility, vascular dysfunction, and a prothrombotic tendency. The PFOCUS study has been designed to systematically investigate these mechanisms and to clarify the relationship between MA and PFO, which may identify a phenotype in which the risk of ischemic stroke and white-matter vascular lesions is attributable to mechanisms beyond paradoxical embolism, opening new perspectives for research and prevention.

## Methods and analysis

### Study design

PFOCUS is a prospective, observational, monocentric, non-pharmacological, no-profit case–control study promoted by and conducted at the IRCCS Institute of Neurological Sciences of Bologna (ISNB). The total planned duration is 42 months (36 months of recruitment and 6 months for data analysis and dissemination). Participants are consecutively recruited and categorized into two groups: cases (RLS-positive), i.e., MA patients with evidence of a right-to-left shunt (RLS) on contrast-enhanced transcranial Doppler (TCD), and controls (RLS-negative), i.e., MA patients without an RLS. This design allows the comparison of biological and physiological characteristics associated with the presence of an RLS within a homogeneous population of patients with MA (Figure 1).

**Figure 1:**
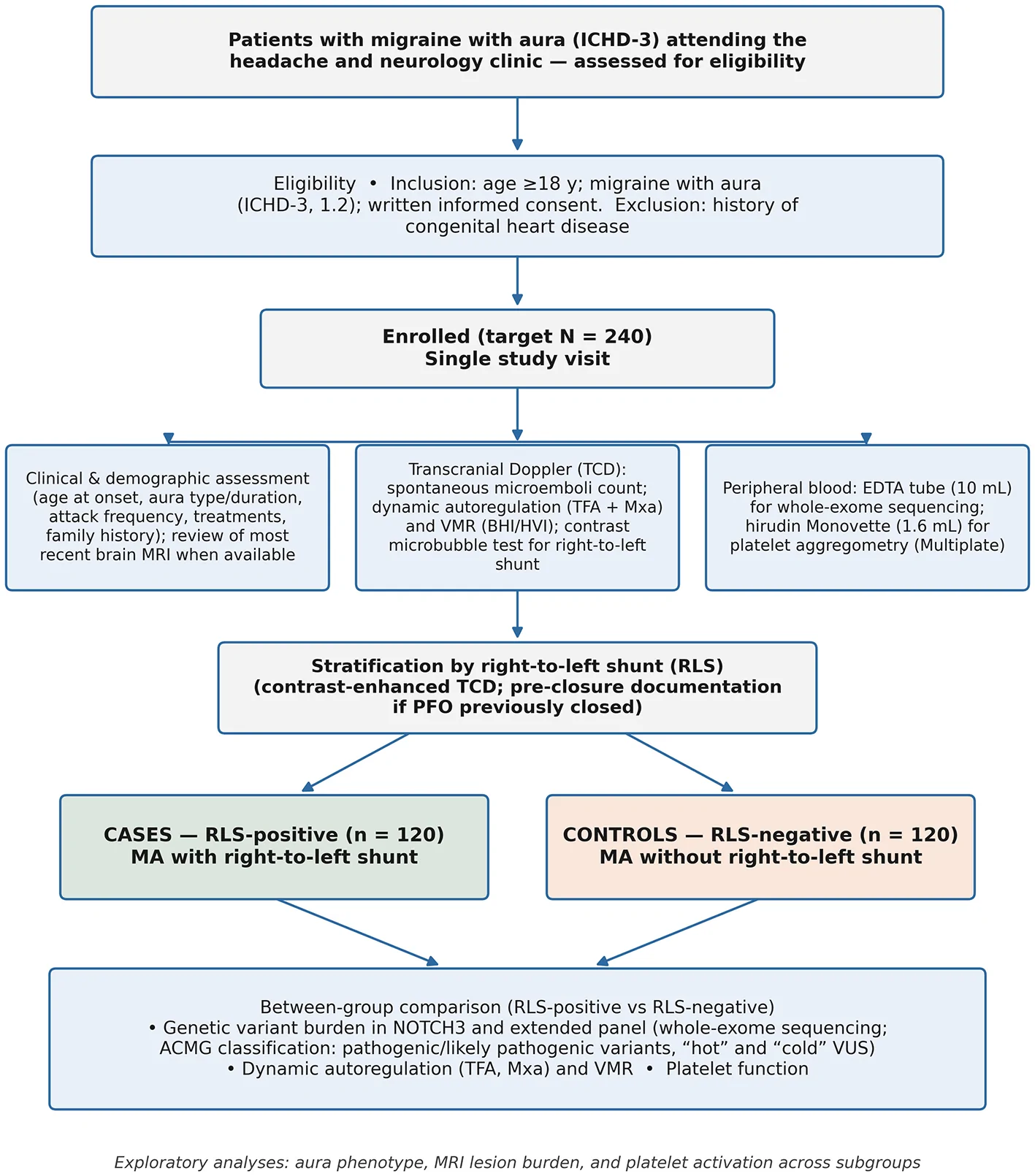
PFOCUS study flow diagram. Patients with migraine with aura (ICHD-3) are assessed for eligibility and, after informed consent, enrolled (target *N* = 240). During a single visit, participants undergo clinical assessment and brain-MRI review, transcranial Doppler (TCD) with microemboli count, dynamic autoregulation and cerebrovascular reactivity assessment and a contrast microbubble test, and peripheral blood sampling for whole-exome sequencing and platelet aggregometry. Participants are stratified by right-to-left shunt into RLS-positive cases (*n* = 120) and RLS-negative controls (*n* = 120) for between-group comparison. VUS, variants of uncertain significance; TFA, transfer function analysis; VMR, vasomotor reactivity; BHI, breath-hold index; HVI, hyperventilation index.

#### Right-to-left shunt as exposure

Group assignment is based on the presence of an RLS on contrast-enhanced TCD, used as a surrogate marker of PFO; patients who have previously undergone percutaneous PFO closure are classified as RLS-positive on the basis of pre-closure documentation. Contrast-enhanced TCD has a pooled sensitivity of 97% and a specificity of 93% for intracardiac RLS compared with transoesophageal echocardiography (TEE) (23), and several studies suggest that its sensitivity may exceed that of contrast TEE (24). TEE was not included as a study procedure because it is semi-invasive, is not clinically indicated in patients with MA without stroke, and would have altered the minimal-risk profile of this non-interventional study. The main source of misclassification is an extracardiac (pulmonary) shunt, which contrast-enhanced TCD cannot distinguish from an intracardiac one. However, pulmonary arteriovenous malformations, the main cause of anatomical extracardiac right-to-left shunting, are rare, with an estimated prevalence of 38 per 100 000 in the general population (25); extracardiac shunts are therefore expected to account for only a small proportion of RLS-positive participants. Throughout the manuscript, RLS-positive and RLS-negative refer to this operational definition, whereas PFO refers to the underlying anatomical hypothesis.

### Study population

The study population consists of adult patients diagnosed with MA according to the International Classification of Headache Disorders, 3rd edition (ICHD-3) (26), consecutively recruited at the participating center (UOC Neurologia OM e Rete Stroke Metropolitana) and at the collaborating unit (Headache and Facial Pain Programme). At the enrolment visit, investigators verify the inclusion and exclusion criteria and provide detailed information about the study; after written informed consent, the patient is considered enrolled.

### Inclusion criteria

- diagnosis of MA (ICHD-3, section 1.2);
- age ≥ 18 years;
- both sexes;
- provision of written informed consent.

### Exclusion criterion

- history of congenital heart disease.

No minimum attack frequency is required for inclusion. Participants are drawn from patients followed at the Headache and Facial Pain Programme, a tertiary referral service which, under the regional care pathway, follows exclusively patients with clinically active primary headache, whereas patients whose headache is no longer active are referred to first-level outpatient services in the community. Participants are also recruited at the UOC Neurologia OM e Rete Stroke Metropolitana, which typically evaluates patients with migraine referred from the emergency department, where they presented because of migraine attacks; in these patients, too, the disease is active at the time of enrolment. Active disease at enrolment is therefore an intrinsic feature of both recruitment routes rather than an applied filter. Disease activity is characterized in every participant (number of attacks in the preceding 3 months, lifetime peak monthly frequency, current and past chronic migraine) and examined in subgroup analyses rather than used to exclude patients, in order to avoid restricting the cohort to the high-frequency phenotype. Likewise, no upper limit for age at aura onset and no minimum disease duration are imposed, since the diagnosis is established by headache specialists according to ICHD-3 criteria, which require that the headache is not better accounted for by another disorder; age at MA onset is recorded, and subgroup and sensitivity analyses by age at onset, including the exclusion of patients with onset at 50 years or later, are planned (Supplementary material 2).

### Sample size

Assuming a two-sided α of 0.05 and 80% power, an anticipated frequency of specific NOTCH3 polymorphisms of 15% in RLS-negative patients with MA and a doubling of this frequency in RLS-positive patients, 120 participants per group (240 in total) are required. The 15% figure, derived from Menon et al. (18), is used as a plausible anchor rather than as a precise estimate of the composite endpoint, which is based on whole-exome data. It is nonetheless consistent with population data: in a Chinese population-based cohort, 13.6% of participants carried at least one rare NOTCH3 variant, defined with the same minor allele frequency threshold (< 1%) adopted in the present study (27). A sensitivity analysis quantifies how the detectable effect varies with the assumed baseline frequency: across baseline frequencies from 10 to 30%, 120 participants per group provide 80% power to detect an odds ratio ranging from approximately 2.8–2.1, whereas power to detect a doubling decreases at lower baseline frequencies (Supplementary material 2). RLS status is determined in all participants (see study procedures); attrition for the primary endpoint is therefore essentially limited to sequencing failure, and power remains at approximately 78% assuming a 5% failure rate. The sample size also reflects the maximum accrual achievable at a single center within the funded 36-month recruitment period; smaller differences will not be reliably detected, and the study is not powered for the secondary endpoints. The 1:1 case-to-control ratio is consistent with the expected prevalence of PFO in patients with MA (approximately 50%).

### Study procedures

Enrolled patients are evaluated during a single study visit. Clinical and demographic data are collected, including age at MA onset, type and duration of aura, concomitant migraine without aura, current and lifetime peak attack frequency, current and previous acute and preventive treatments, medical history, and family history of cerebrovascular disease, cerebral hemorrhage, or dementia; variables may also be retrieved from the electronic medical record. The full list of collected variables is provided in Supplementary material 2.

All assessments are performed in the interictal state, operationally defined as the absence of headache and aura at the time of the study visit and the absence of any attack on the day of the assessment, verified by the investigator at enrolment. Participants are observed for 1 h after the contrast study, and any migraine attack occurring during this period is recorded. The number of days since the last migraine attack and the interval from the last intake of acute medication are recorded. Current acute treatments (including non-steroidal anti-inflammatory drugs [NSAIDs] taken in the preceding 48 h), current and previous preventive treatments by drug class with their response, and ongoing antiplatelet and estrogen–progestogen therapy are documented. Because antiplatelet agents, NSAIDs and some preventive drugs (e.g., antidepressants) may affect platelet aggregation, and vasoactive preventive drugs (e.g., calcium-channel blockers and beta-blockers) may affect cerebral hemodynamics, the primary platelet-function comparison is restricted to participants not exposed to antiplatelet agents or NSAIDs, and the haemodynamic and platelet analyses include sensitivity analyses adjusted for, or stratified by, these treatment classes (Supplementary material 2).

Patients then undergo a neurosonological study with transcranial Doppler (TCD), comprising:

- automated spontaneous cerebral microemboli count (Dolphin 4D);
- assessment of dynamic cerebral autoregulation (dCA) and cerebral vasomotor reactivity (VMR) by breath-holding and hyperventilation tests;
- contrast microbubble test for right-to-left shunt detection.

For dCA, cerebral blood flow velocity (CBv) in the middle cerebral arteries (evaluated by bilateral robotic TCD) and beat-to-beat arterial blood pressure (ABP, servo-controlled finger plethysmography/finometer) are co-recorded continuously for approximately 10 min with the patient at rest, from which stable, artifact-free segments of at least 5 min are extracted for analysis. VMR is assessed with the same setup after 30 s of apnea (breath-holding test) and 30 s of hyperventilation. Technical details on dCA and VMR registration and analysis are reported in Supplementary material 1.

The microbubble test involves the infusion of agitated saline through a peripheral venous access during Doppler monitoring. At the time of peripheral catheter placement, two blood samples are collected: one for platelet aggregometry (hirudin Monovette^®^ tube, 1.6 ml; analyzed with the Multiplate^®^ system at the Laboratorio Unico Metropolitano, LUM) and one for genetic analysis (EDTA tube, 10 ml). In participants without an adequate transtemporal acoustic window, dCA and VMR are not assessed, and the microbubble test is performed with transcranial color-coded duplex sonography (TCCD) through the transforaminal (suboccipital) window, so that RLS status is determined in all participants.

#### Brain MRI

When available, the most recent brain MRI is retrieved from the institutional PACS or, if unavailable, from a patient-provided CD. Scans are included in the imaging analyses if they meet the following minimum requirements: field strength ≥1.5 T and an axial fluid-attenuated inversion recovery (FLAIR) or T2-weighted sequence with slice thickness ≤ 5 mm; microbleeds and superficial siderosis are rated only when a T2^*^-weighted gradient-echo or susceptibility-weighted sequence is available, and are otherwise recorded as not assessable. Scans are rated centrally by an expert neuroradiologist (S.S.) blinded to shunt status and clinical data. White-matter hyperintensities are graded with the Fazekas scale, separately for deep and periventricular white matter (28), and the other markers are defined according to the STRIVE-2 standards (29): lesions in the anterior temporal poles, external capsules and cerebellum, previous ischaemic or haemorrhagic lesions, and microbleeds or superficial siderosis. To assess reliability, a random sample of 20% of scans is independently rated by a second neuroradiologist, and inter-rater agreement is quantified with the weighted kappa statistic. Field strength, available sequences and the interval between the scan and the study visit are recorded for each examination.

### Genetic analysis

The Neurogenetics Laboratory of the IRCCS ISNB performs the genetic analyses by whole-exome sequencing (WES), from which information is extracted for a broad first-level gene panel covering Notch signaling, cardiac septation and PFO development, and cerebral small-vessel disease, including: NOTCH3, NOTCH1, JAG1, HTRA1, GATA4, TBX20, TBX5, NKX2-5, PITX2, ACTC1, MYH6, ATP1A2, EFEMP1, LTBP1, LDLR, SDHC, ABCC6, ABCG2, ACE, AKAP9, AKT2, APOB, CACNA1A, CELA2A, CITED2, CLCN2, CNOT2, COL4A1, DMD, DSC2, ELN, EPAS1, EPOR, ESR1, EYA4, FLNC, FLT4, FRG1, HCCS, IGBP1, KCNMB4, LAMA4, LRP6, MLLT10, MYBPC3, MYH11, PRDM16, PSEN2, RNF213, RYR2, SCN5A, SCNN1B, SDHD, TNNI3K, VCL, WBP11, G6PC1, SERPINA1, FOXC1, and EPHX2 (12, 30, 31). Libraries are prepared by enzymatic fragmentation (xGen DNA EZ Library Prep Kit, IDT), enriched with the xGen Exome Research Panel v2 (IDT), which targets coding regions and exon–intron junctions (±100 bp), and sequenced paired-end (2 × 100 bp) on a NovaSeq 6,000 platform (Illumina). Reads are aligned to the hg38 reference genome with BWA (v0.7.17), and variants are called in multi-sample mode with GATK (v4.2.0.0) and annotated with Ensembl VEP (v109.3), dbNSFP (v4.3a) and dbscSNV (v1.1). For research purposes, the complete variant call set is analyzed, irrespective of the filters applied in clinical reporting. Identified variants are classified according to the criteria of the American college of medical genetics and genomics (ACMG) (32), distinguishing pathogenic/likely pathogenic mutations from variants of uncertain significance (VUS), the latter further subdivided into “hot” and “cold” VUS for the purposes of the registered primary endpoint, according to the laboratory classification used in the original protocol; operationally, “hot” and “cold” VUS correspond, respectively, to the “VUS – leaning pathogenic” and “VUS – leaning benign” categories generated by the Franklin interpretation framework (Genoox, now QIAGEN; https://franklin.genoox.com). This subclassification is not an ACMG category and is used only to define the registered endpoint reproducibly. Variant interpretation is performed blinded to RLS status and relies on gnomAD (release r4.0.0) (33), ClinVar (34), PanelApp, the published literature and the VarSome and Franklin platforms. Qualifying variants are rare (minor allele frequency < 1% in the gnomAD non-Finnish European population) and protein-altering or affecting canonical splice sites. Pathogenic and likely pathogenic variants are confirmed by Sanger sequencing (ABI 3500dx), and all software and database versions, including the Franklin version and date of access, are documented. For the primary analysis, the same pre-defined classification procedure is applied to all participants using a single database and software release (data freeze at the time of analysis), so that classification does not depend on the date of sequencing; any subsequent reclassification is reported separately.

At enrolment, patients may choose whether to receive information about the results of the analyses, and are informed of the possibility of detecting pathogenic mutations, in particular for CADASIL. When a pathogenic mutation is identified and the patient has chosen to be informed, genetic counseling is provided. The communication of incidental findings follows the guidelines of the European society of human genetics (ESHG), which recommend reporting only mutations associated with serious health problems for which early diagnosis or effective treatment is possible.

### Endpoints

#### Primary endpoint

Difference in the frequency of NOTCH3 gene variants (pathogenic/likely pathogenic mutations and “hot”/“cold” VUS, classified per ACMG criteria) between RLS-positive and RLS-negative patients with MA.

#### Secondary endpoints

- difference in the frequency of pathogenic/likely pathogenic NOTCH3 variants alone between groups;
- differences in the frequency of variants in the other selected panel genes between groups;
- correlation between variant frequency and right-to-left shunt size in the RLS-positive group;
- differences in cerebral microemboli count between groups;
- differences in VMR and in dCA parameters between groups;
- differences in platelet function and prothrombotic profile between groups.

#### Exploratory endpoints

- association between aura characteristics (e.g., age at onset, visual vs. sensory aura, duration, frequency) and haemodynamic parameters;
- differences in platelet activation profiles across aura subtypes;
- correlation between specific genetic variants and distinct aura phenotypes;
- differences in the presence and distribution of small vessel disease or vascular lesions on MRI.

### Statistical analysis

Statistical analyses will be performed with R software. Demographic variables of the two groups will be compared with parametric or non-parametric tests, as appropriate. A detailed statistical analysis plan is provided in Supplementary material 2; its revised version (v1.1), finalized before database lock and before any analysis of study outcomes, distinguishes the analyses pre-specified in the original plan from the secondary, subgroup and sensitivity analyses.

For variants identified by exome sequencing, the frequency of each variant and the overall frequency of “hot” and “cold” VUS and of pathogenic mutations (defined according to ACMG criteria) will be computed in both groups. Between-group differences (RLS-positive vs. RLS-negative) will be assessed with the chi-square test or Fisher's exact test, depending on cell counts. As a complementary single-nucleotide-polymorphism (SNP)–level analysis, genotypes will be coded according to the minor allele (0 for major-allele homozygotes, 1 for heterozygotes, 2 for minor-allele homozygotes); allele and genotype distributions will be compared between groups using contingency tables and the chi-square test. All SNPs will be tested for Hardy–Weinberg equilibrium, and linkage disequilibrium will be evaluated with dedicated software.

The primary endpoint and the key secondary analysis restricted to pathogenic/likely pathogenic NOTCH3 variants are single comparisons, tested at a two-sided α of 0.05 without adjustment. For the other secondary and complementary analyses, the false discovery rate will be controlled with the Benjamini–Hochberg procedure (*q* = 0.05) (35) within pre-defined families of tests: (i) gene-level comparisons across the remaining panel genes; (ii) SNP-level association tests; (iii) haemodynamic parameters (microemboli, dCA and VMR indices); and (iv) platelet-function parameters. Exploratory analyses will not be adjusted for multiplicity; they will be reported with effect sizes, 95% confidence intervals and the number of tests performed, and interpreted as hypothesis-generating.

Dynamic cerebral autoregulation will be quantified in both the frequency domain and the time domain. In the frequency domain, transfer function analysis (TFA) will be applied to the CBv–ABP relationship to estimate coherence, gain, and phase shift within the very-low-frequency (0.02–0.07 Hz), low-frequency (0.07–0.2 Hz), and high-frequency (0.2–0.5 Hz) ranges, following the CARNet white paper recommendations (36). In the time domain, autoregulation will be assessed using the moving correlation coefficient Mx—specifically Mxa, the correlation between spontaneous fluctuations of mean ABP and mean CBv—in accordance with the CARNet time-domain recommendations (37). VMR will be expressed as the breath-holding index (BHI) for the apnoea test and the hyperventilation index (HVI) for the hyperventilation test.

### Data management

Data are collected in a pseudonymised format, using a unique alphanumeric code, in a secure electronic database that meets adequate anonymity and data-protection requirements. Demographic and clinical data, treatment history, family history, and neuroimaging obtained as part of routine care are recorded on a paper case report form and transcribed into the electronic database. Patient data and biological samples are identified by the same unique code, used only for study purposes. Biological samples are stored in separate containers in controlled, locked rooms and subsequently sent to the Neurogenetics Laboratory of the IRCCS ISNB; samples may be used for a maximum of 10 years (unless the patient withdraws consent earlier), after which they are destroyed.

## Discussion

### Expected impact

PFOCUS is designed to move the interpretation of the migraine–PFO association beyond the paradoxical embolism paradigm. By combining, within the same patients and during a single visit, whole-exome-based variant analysis, dynamic cerebral autoregulation and vasomotor reactivity, and platelet function testing, the study will provide the first integrated characterization of MA patients stratified by right-to-left shunt. Should RLS-positive patients show a higher burden of NOTCH3 variants, this would support the hypothesis that incomplete foramen ovale closure and cerebral small-vessel dysfunction share a common developmental substrate in Notch signaling, rather than co-occurring by chance. Conversely, a neutral genetic result with divergent haemodynamic or platelet profiles would still be informative, pointing toward acquired rather than constitutional determinants of the association. Either outcome would refine the biological plausibility of a distinct MA-plus-PFO phenotype and inform the ongoing debate on which patients with MA, if any, may derive benefit from PFO closure.

### Limitations

Several limitations should be acknowledged at the design stage. First, PFOCUS is a monocentric study conducted at a tertiary headache and stroke center, which may favor the recruitment of patients with more frequent or complex aura and limit generalisability. This is an intrinsic consequence of the regional care pathway, under which only patients with active and clinically complex primary headache are managed at tertiary level; the cohort is therefore expected to be enriched for high-burden phenotypes, and frequency estimates derived from it should not be extrapolated to the general population with migraine. Second, the case–control design with cross-sectional phenotyping permits the identification of associations but not of causal relationships; while genetic variants are constitutional and therefore not subject to reverse causation, haemodynamic and platelet measures reflect a single interictal time point and may be influenced by prophylactic or antiplatelet treatment, which is addressed analytically as described above. Moreover, no fixed minimum interval from the last attack was imposed *a priori*, so that a proportion of measurements may fall within the postdromal phase; the interval from the last attack, where available, and from the last intake of acute medication will be reported and examined in sensitivity analyses. Third, the sample size is powered for the primary genetic endpoint only, and power would be lower than planned if the baseline frequency of qualifying NOTCH3 variants were below 10%; secondary analyses are not formally powered, and exploratory findings must be regarded as hypothesis-generating. Fourth, the classification of variants of uncertain significance is dynamic, and the distinction between “hot” and “cold” VUS, although based on a standardized classification platform, is not a formal ACMG category; reclassification over time may modify the composition of the primary outcome. For this reason, pathogenic/likely pathogenic variants are also analyzed separately, and variant interpretation will be documented with the reference databases and versions used. Fifth, neuroimaging is not standardized within the study. Brain MRI is not performed as a study procedure: the analysis of white-matter lesion burden and distribution relies on the most recent clinically indicated scan, retrieved from the institutional PACS or provided by the patient. Consequently, MRI data will be unavailable for a proportion of participants, introducing potential selection bias, since patients who have undergone neuroimaging may differ systematically from those who have not — for example in aura complexity, disease duration, or clinical concern raised during previous assessments. Moreover, the available examinations will have been acquired on different scanners, with heterogeneous field strengths, sequences and slice thickness, and at variable intervals from the study visit. This limits the comparability of lesion assessment across participants and confines the imaging analyses to a strictly exploratory role. To mitigate these constraints, only scans meeting defined minimum technical requirements will be analyzed, all scans will be rated centrally by a neuroradiologist blinded to shunt status using the Fazekas scale and STRIVE-2 definitions rather than volumetric quantification, inter-rater agreement will be assessed on a random subsample, and the proportion of participants with usable MRI, together with scanner field strength and time from the study visit, will be reported. Sixth, exome sequencing detects single-nucleotide variants with a sensitivity above 99% at sites with a read depth of at least 20 ×, but is less sensitive for small insertions/deletions and in technically challenging regions, and does not detect copy-number or structural variants, large insertions/deletions, repeat expansions or low-level mosaicism; the impact on NOTCH3 analyses is expected to be limited, since the variants underlying CADASIL are predominantly missense. Finally, shunt ascertainment relies on contrast-enhanced transcranial Doppler, which is highly sensitive for right-to-left shunt but does not discriminate an interatrial from an extracardiac (pulmonary) shunt in the absence of transoesophageal echocardiography; a proportion of misclassification is therefore possible, although it is expected to be small given the rarity of pulmonary arteriovenous malformations (25).

### Ethics and dissemination

Participation in the study entails minimal risk: peripheral venous cannulation, agitated-saline contrast injection and breath-holding and hyperventilation maneuvers are low-risk procedures routinely performed in clinical practice. The study is conducted in accordance with the principles of Good Clinical Practice (ICH-GCP) and the Declaration of Helsinki. The present protocol (version 1, 14th March 2025) received a favorable opinion from the Ethics Committee Area Vasta Emilia Centro (CE AVEC; reference 293-2025-TESS-AUSLBO; local study code CE25057) on 28 October 2025 and was subsequently authorized by the Institutional Direction of the IRCCS ISNB. Written informed consent is obtained from all participants before enrolment. The study was registered at ClinicalTrials.gov (NCT07349004) in January 2026.

Any protocol modification will be implemented as a formal amendment. The investigators undertake to disseminate the results within 1 year of study completion through publication in peer-reviewed journals, with patient data reported in aggregate and anonymised form.

## Current study status

The study is ongoing, with recruitment active since November 2025. At the time of writing, enrolment is in progress toward the planned target of 240 participants. The planned 42-month timeline, with the current time point indicated, is shown in Figure 2.

**Figure 2:**
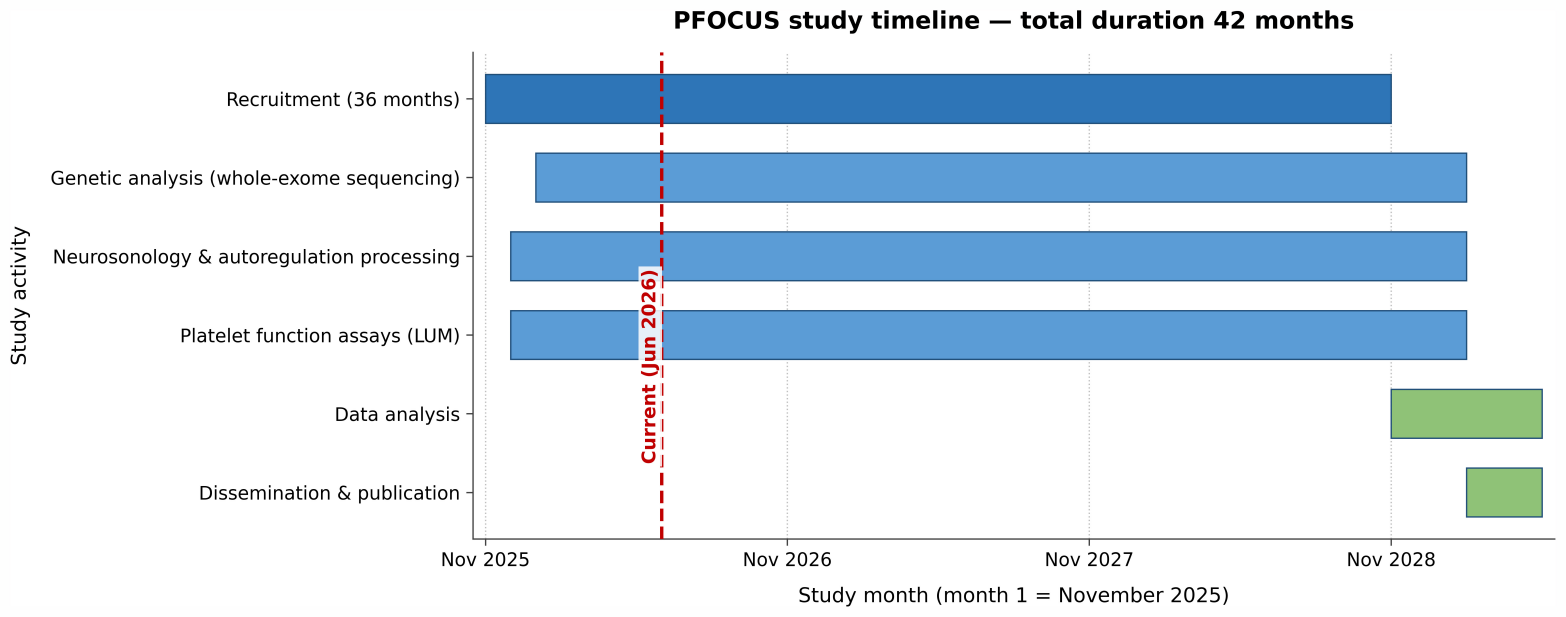
PFOCUS study timeline. Gantt chart of the planned 42-month study (36 months of recruitment plus 6 months of analysis and dissemination). Month 1 corresponds to November 2025. The dashed red line marks the current time point (June 2026); recruitment has been active since November 2025.

## Conclusion

PFOCUS will provide a multimodal, genetically informed characterization of migraine with aura in the presence and absence of a right-to-left shunt. By integrating variant burden in the Notch pathway and in genes implicated in cardiac septation and cerebral small-vessel disease with measures of cerebral autoregulation, vasomotor reactivity and platelet function, the study aims to establish whether MA with PFO constitutes a biologically distinct phenotype. If confirmed, such a phenotype could contribute to stroke risk stratification in young patients with migraine and support the development of precision-medicine approaches to prevention. Recruitment is ongoing, and the results are expected to be disseminated through peer-reviewed publication within 1 year of study completion.
