# Supplementary material for: PFOCUS study protocol: a prospective case–control study investigating genetic, haemodynamic, and platelet function in migraine with aura with or without patent foramen ovale
Source: Front Neurol. 2026 Oct 16;17:1952744. doi: 10.3389/fneur.2026.1952744 (PMC13719609; doi:10.3389/fneur.2026.1952744)
Supplement: Supplementary file 1 [file Table_1.docx]

**Supplementary Material 1**

*Supplementary Material 1 to the PFOCUS study protocol — Neurosonological acquisition and signal-processing methods*

**Part A — Acquisition and signal-processing methodology for dynamic cerebral autoregulation (dCA) analysis**

| **Item** | **Value** |
| --- | --- |
| Analysis software | dca-autoreg — original R implementation of the CARNet transfer function analysis and time-domain framework |
| Software version | v1.11 (processing specification v1.11; curation schema v4) |
| Runtime | R 4.3.3 |
| Corresponding author | Matteo Paolucci, IRCCS Istituto delle Scienze Neurologiche di Bologna / AUSL Bologna, Italy |

# 1 Purpose and scope

Part A of this supplementary material documents, at a level of detail sufficient for independent reimplementation, the acquisition set-up and the complete signal-processing chain used to derive dynamic cerebral autoregulation (dCA) indices in the PFOCUS study. It is intended to satisfy the reporting requirements of the CARNet white papers (1, 2) and of the INfoMATAS methodological review (3), which identify incomplete reporting of processing settings as the principal obstacle to comparability between dCA studies.

The document describes *what the analysis does* and *with which parameter values*, not how it is implemented in code. Every processing decision not fixed by the CARNet recommendations is stated explicitly with its rationale, so that the effect of each local choice can be assessed by the reader. Deviations from the recommendations are identified as such rather than presented as compliance.

# 2 Signal acquisition

Recordings were obtained with the patient supine, at rest and in a quiet environment, during spontaneous blood-pressure fluctuations; no provocation manoeuvres were used.

| **Parameter** | **Value** |
| --- | --- |
| CBFV device | Viasonix Dolphin 4D, robotic headframe, 2 MHz probes |
| Insonation | Bilateral middle cerebral artery, depth 50–60 mm, 12 mm sample |
| ABP device | Finapres NOVA — Penáz volume-clamp method, double finger cuff on the intermediate phalanx of the third digit |
| ABP level correction | Height Correction Unit (hydrostatic column) active throughout |
| ABP channel used | reBAP (reconstructed brachial arterial pressure), calibrated against an upper-arm cuff before acquisition, with periodic recalibration |
| Native sampling frequency | Finapres NOVA 200 Hz; Viasonix Dolphin 4D 125 Hz |
| Analysed record | Both signals are analysed from the 125 Hz Viasonix export; the fixed inter-device offset of 4 s (section 3.1) corresponds to 500 samples at this rate |
| Anti-aliasing filter | 20 Hz low-pass, applied in hardware by the acquisition devices (REC #4); no software anti-aliasing step is applied |
| Minimum recording duration | 5-10 min of analysable signal (REC #3) |
| Export format | Raw CSV, semicolon-separated, dot decimal separator, UTF-8 with BOM |
| Additional channels | ECG; nasal capnogram (end-tidal CO₂), when obtainable |

Supplementary Table S1. Acquisition parameters.

**Note on file format.** The raw CSV export is used rather than the device spreadsheet export because, under an Italian system locale, spreadsheet software misinterprets dot decimal separators in the ECG and capnogram columns as thousands separators, silently corrupting those channels. All analyses read the raw CSV directly.

# 3 Pre-processing

## 3.1 Inter-device synchronisation

The Finapres NOVA output is delayed by a fixed 4 seconds interval relative to the Viasonix TCD stream. This delay is a property of the device pairing rather than of the individual acquisition.

Correction is applied at the extraction stage by offsetting the ABP window (exported at 125 Hz) by 500 samples relative to the CBFV window, rather than by shifting either signal after beat-to-beat conversion.

**Reporting implication.** Any group replicating this protocol with a different device combination must characterise its own inter-device latency. We recommend that studies combining a finger-cuff pressure device with a separate TCD system report this measurement explicitly, since an uncorrected offset of this magnitude is sufficient to reverse the direction of a phase-based conclusion.

## 3.2 Cardiac cycle detection

Beat detection uses the ECG R-wave as the primary fiducial, with a two-threshold Schmitt trigger: a candidate is entered when the signal exceeds 50% of the adaptive amplitude reference and closed when it falls below 80% of that reference, with a maximum growth constraint of two samples. The hysteresis prevents fragmentation of single complexes into multiple detections in the presence of amplitude drift.

When the ECG channel is absent or drops out, detection falls back to a pulse-based algorithm operating on the CBFV waveform. Gaps in ECG availability are filled hybridly, so a single recording may contain both ECG-derived and pulse-derived markers; the provenance of each marker is retained in the analysis metadata.

**Phantom markers.** T-wave over-detection produces spurious markers offset by approximately 0.3 s and coinciding with the ABP systolic peak. These are detected automatically, flagged separately from physiological artefacts, and *deleted* rather than interpolated, since they represent a detection failure and not a missing beat.

## 3.3 Beat-to-beat series extraction

Beat-to-beat mean ABP and mean CBFV are obtained by waveform integration (area under the curve) over each cardiac cycle, in accordance with REC #5. Each value is time-stamped at the ABP systolic peak of the corresponding cycle.

**Deviation from REC #5.** The white paper suggests anchoring the cardiac cycle at the ABP diastolic value. We time-stamp at the systolic peak because the peak is more robustly identifiable in the presence of the reBAP reconstruction and of the Physiocal calibration transients described below. Because the same convention is applied to both signals, the relative timing that determines transfer function phase is unaffected; only the absolute time axis is displaced by a sub-beat interval, far below the frequency resolution of the analysis.

Mean CBFV per cycle is used as the output signal.

## 3.4 Physiocal calibration segments

The Finapres Physiocal algorithm periodically interrupts the volume-clamp servo to re-derive the set-point, producing recurring short segments (typically 1–3 s, recurring every 30–70 s) during which the ABP waveform is not physiological. These are the most common source of ABP contamination in bedside recordings.

Detection uses rolling variance of the ABP signal with an asymmetric buffer (1.2 s before, 0.5 s after the detected block) and the same two-threshold hysteresis logic used for beat detection, so that the gradual recovery tail following a calibration is captured rather than truncated. The first and last 3 s of the recording are excluded from detection, to avoid mistaking initial servo stabilisation for a Physiocal event, and blocks longer than 4 s are rejected as implausible.

Detected Physiocal segments are replaced by interpolation within the continuous beat-to-beat series; the series is never cut and re-concatenated around them. Excision-and-concatenation introduces a phase discontinuity at every join and broadband spectral leakage, contaminating precisely the low-frequency range on which dCA estimates depend. This applies regardless of segment length and constitutes a pathway distinct from the general artefact rules in section 3.5.

## 3.5 Artefact handling and missing data

Artefacts are annotated per channel — ABP, left CBFV, right CBFV, or all channels simultaneously — so that unilateral probe loss does not invalidate the contralateral estimate or the pressure signal. Annotation is performed in a dedicated review interface by an operator inspecting the raw traces. An automated artefact advisor overlays candidate regions but is advisory only and is never applied without operator confirmation.

| **Situation** | **Rule applied** |
| --- | --- |
| Contaminated run ≤ 3 beats | Linear interpolation across the affected beats in the beat-to-beat series |
| Contaminated run > 3 beats | Retained as a genuine missing-data gap (NA) on the series copy that feeds TFA; no interpolation |
| Physiocal segment (any length) | Interpolated in place — separate pathway, see section 3.4 |
| Phantom (T-wave) markers | Deleted; excluded from the artefact count and from interpolation |
| Welch segment containing a gap | The whole segment is discarded from spectral averaging, and the coherence critical value is recalculated from the number of surviving segments |

Supplementary Table S2. Artefact and missing-data handling rules.

**Rationale for the last rule.** Interpolating across long gaps to preserve segment count would keep the nominal number of Welch windows high and thereby keep the coherence critical value artificially low, making the resulting estimates appear more reliable than the data support. Discarding the segment and re-deriving the threshold from the surviving segments makes the loss of statistical power explicit rather than concealing it.

## 3.6 Ectopic beats

Ventricular extrasystoles and conducted atrial extrasystoles are retained for the dCA analyses (transfer function analysis and Mx). Following the CARNet 2022 supplementary material, an ectopic beat is treated as a spontaneous physiological pressure challenge, and its removal would discard genuine autoregulatory information.

The same beats are corrected for the autonomic analyses (heart rate variability and baroreflex sensitivity), where the contaminated RR interval and systolic value are non-physiological by construction and would bias the derived indices. Correction is applied to the RR and systolic series only, on a separate copy, and is guarded by an atrial-fibrillation check that disables ectopic correction when the rhythm is irregularly irregular.

## 3.7 Resampling

Beat-to-beat series are resampled onto a uniform time grid at 10 Hz by linear interpolation, exceeding the 4 Hz minimum of REC #7. No detrending, normalisation or filtering is applied to the resampled data (REC #8, #9).

# 4 Transfer function analysis

Transfer function analysis uses mean ABP as input and mean CBFV as output, with auto- and cross-spectra estimated by the Welch method. Supplementary Table S3 lists every setting alongside the corresponding CARNet 2022 recommendation.

| **Setting** | **CARNet 2022 (Table 1)** | **Adopted here** | **REC #** |
| --- | --- | --- | --- |
| Input data | Beat-to-beat | Beat-to-beat | 5 |
| Minimum duration | 5 min | 5 min | 3 |
| Raw sampling frequency | ≥ 50 Hz | 125 Hz (analysed record) | 4 |
| Anti-aliasing low-pass | 20 Hz | 20 Hz, device-side | 4 |
| Re-sample frequency | ≥ 4 Hz | 10 Hz | 7 |
| Interpolation | Linear | Linear | 7 |
| Detrending | None | None | 8 |
| Normalisation | None | None | 8 |
| Filtering of resampled data | None | None | 9 |
| Anti-leakage window | Hanning | Hanning | 10 |
| Window length | 100 s | 100 s | 11 |
| Superposition (overlap) | 50% | 50% | 11, 12 |
| Spectral smoothing | Triangular (¼, ½, ¼) | Triangular (¼, ½, ¼) | 13 |
| Coherence threshold | 95% confidence limit | 95% limit, 2022 Table 2 | 14 |
| Frequency bands (Hz) | VLF 0.02–0.07; LF 0.07–0.20; HF 0.20–0.50 | Identical; lower limit inclusive, upper limit exclusive | 13 |
| Phase units | Degrees or radians | Degrees and radians | 15 |
| Gain units | Absolute and normalised | Both reported | 15 |

Supplementary Table S3. Transfer function analysis settings, mapped to the CARNet 2022 recommendations.

## 4.1 Coherence thresholding

The critical value for magnitude-squared coherence depends on the number of Welch segments and is therefore recomputed for every recording. A fixed threshold — such as the commonly used 0.5 — is not used, since CARNet 2022 explicitly warns that it causes both erroneous inclusion and erroneous rejection of frequencies. Values are taken from Table 2 of Panerai et al. (2) at the 95% level.

| **Segments** | **3** | **4** | **5** | **6** | **7** |
| --- | --- | --- | --- | --- | --- |
| Critical value | 0.51 | 0.41 | 0.34 | 0.29 | 0.25 |
| **Segments** | **8** | **9** | **10** | **11** | **12** |
| Critical value | 0.22 | 0.20 | 0.18 | 0.17 | 0.15 |
| **Segments** | **13** | **14** | **15** | **20** | **25** |
| Critical value | 0.14 | 0.13 | 0.12 | 0.09 | 0.08 |

Supplementary Table S4. Coherence critical values at the 95% level as a function of the number of Welch segments (2, Table 2). For segment counts that are not tabulated, the implementation interpolates linearly between adjacent entries and clamps outside the tabulated range.

**Version of the table.** The 2022 values are used throughout. The 2016 white paper reports slightly different Monte Carlo values for the same segment counts; mixing the two across a literature comparison introduces a systematic bias in the number of frequencies retained, and the version used should always be stated.

## 4.2 Treatment of the VLF band

Coherence is computed across all frequency bins irrespective of threshold. For the LF and HF bands, band-average gain and phase are computed over bins exceeding the critical value, per REC #14. For the **VLF band**, gain and phase are reported without coherence thresholding. This is a deliberate deviation from REC #14.

**Rationale.** Below 0.07 Hz the pressure–flow relationship is intrinsically non-linear, because of large time-varying changes in arterial diameter. Coherence in this band is therefore reduced by the physiology itself and not only by noise, and thresholding removes preferentially those frequencies at which autoregulation is most active. Correspondingly, low individual VLF coherence values are not interpreted as evidence of poor data quality and are not used as a primary argument in any clinical inference.

## 4.3 Phase handling

Phase estimates are subject to wrap-around, which in the VLF band manifests as implausible positive values clustering around 120–127°. Negative phase values below 0.1 Hz are removed, and values in the anomalous range are flagged automatically for review rather than silently retained or silently discarded. The frequency of such flags is reported with the results.

## 4.4 Absolute and normalised gain

Gain is reported both in absolute units (cm·s⁻¹·mmHg⁻¹) and normalised to the mean CBFV of the same channel (%·mmHg⁻¹, computed as gain divided by mean CBFV × 100), as required by REC #15.

## 4.5 Spectral reporting

For each recording, gain, phase and coherence are plotted over the full 0.02–0.5 Hz range with across-segment standard deviation bands and the coherence critical value shown as a horizontal reference line, satisfying REC #15 and #17. Phase standard deviation is computed with wrap-safe alignment across segments. A standard deviation of exactly zero indicates that a single segment survived in that band and is flagged as such.

# 5 Time-domain analysis: the correlation index

The correlation index is computed as the Pearson correlation between averaged ABP and averaged CBFV over consecutive short epochs, following the CARNet time-domain white paper (4). Because intracranial pressure is not measured, the index computed is strictly *Mxa* — based on arterial pressure rather than cerebral perfusion pressure. This is stated explicitly wherever the index is reported, since the two are not interchangeable and the distinction is frequently blurred in the literature. Sx and Dx (systolic and diastolic variants) are computed in parallel.

| **Parameter** | **Value** |
| --- | --- |
| Block length, epoch length and overlap | 3s-60s-no overlap; 10s-300s-no overlap; 10s-300s-60s |
| Input pressure signal | Mean ABP (Mxa); cerebral perfusion pressure not available |
| Effective frequency range captured | < 0.1 Hz |
| Interpretation | +1 = pressure-passive circulation; 0 = intact autoregulation; negative values are not physiologically interpretable and are reported as observed |

Supplementary Table S5. Correlation index computation parameters.

**Limitations acknowledged.** Mxa is sensitive to epoch length and to the number of epochs entering each correlation, and its test–retest reliability in short recordings is limited (5). Both the epoch length and the epoch count are therefore reported, and values are interpreted as continuous descriptors rather than dichotomised against a threshold.

# 6 Ancillary outcome measures

REC #17 requires that dCA estimates be reported alongside the physiological context in which they were obtained. The following are computed and exported for every recording:

- Mean arterial pressure; minimum and maximum ABP over the analysed window
- Mean CBFV per side; systolic, mean and diastolic means
- Mean end-tidal CO₂ and its variability, when a capnogram is available; the pipeline runs without a capnogram and records its absence explicitly rather than substituting a default value
- Blood pressure and CBFV variability: standard deviation, coefficient of variation, average real variability, successive variation
- Spectral power of ABP and of CBFV in each frequency band
- Heart rate variability and baroreflex sensitivity, computed on the ectopic-corrected series (section 3.6)
- Non-invasive intracranial pressure estimate and pulsatility index

All of the above are exported to recording-level and batch-level CSV files together with the analysis settings actually used, so that every reported value can be traced back to the parameter set that produced it.

# 7 Quality control and exclusion criteria

Quality is assessed at recording level and recorded as an explicit flag, propagated to every downstream export.

| **Flag** | **Meaning and consequence** |
| --- | --- |
| OK | No quality concern identified; included in primary analyses |
| WARNING | A specific concern is documented (missing contralateral channel, band below coherence threshold, single surviving Welch segment); included, with the concern reported |
| LOW_QUALITY | Signal quality judged poor at acquisition or at review; retained in the dataset, excluded from primary analyses, used in sensitivity analyses only |
| EXCLUDE | Not analysable; retained in the participant flow diagram for STROBE accounting |

Supplementary Table S6. Quality control flags.

**Automatic low-quality suggestion.** A recording is automatically proposed for LOW_QUALITY when the proportion of the analysed window affected by artefacts or gaps exceeds 10%. The suggestion is advisory; the final flag is assigned by the operator.

**Exclusion for persistently low coherence.** In line with REC #14, a recording in which coherence fails to reach significance across all frequency bands is excluded from transfer function analysis, since gain and phase are then uninterpretable. This criterion is applied to LF and HF; it is not applied on the basis of VLF coherence alone, for the reasons set out in section 4.2.

**Step-response plausibility check.** The step response derived from the inverse transform of the impulse response is inspected as a quality control: absence of a clear initial rise, large negative excursions, a continuously rising response, or large oscillations within the first 10 s are treated as indicators of an unreliable transfer function estimate (REC #18).

# 8 Software verification

## 8.1 Verification of the spectral engine

The spectral core of the pipeline was verified against the calibration dataset distributed with the CARNet reference implementation, for which expected values are published in Table 3 of Claassen et al. (1). The dataset was processed with the settings of Supplementary Table S3 (5 segments, 50% overlap, coherence critical value 0.34).

| **Band** | **Gain abs. expected** | **Gain abs. obtained** | **Gain norm. expected** | **Gain norm. obtained** | **Phase (°) expected** | **Phase (°) obtained** |
| --- | --- | --- | --- | --- | --- | --- |
| VLF | 0.68 | 0.676 | 1.04 | 1.041 | 53.0 | 52.97 |
| LF | 0.96 | 0.958 | 1.48 | 1.475 | 25.4 | 25.44 |
| HF | 1.20 | 1.199 | 1.85 | 1.846 | 9.38 | 9.38 |

Supplementary Table S7. Verification of the spectral engine against the CARNet calibration dataset (left channel). Agreement to three significant figures for every band and every outcome measure.

## 8.2 Scope and limits of this verification

The scope of this exercise should be stated plainly. It verifies the **spectral engine** — windowing, Welch averaging, spectral smoothing, cross-spectral estimation, band averaging and coherence thresholding — because the CARNet calibration dataset is supplied already in beat-to-beat, resampled form.

It does *not* verify the upstream chain: import, beat detection, Physiocal handling, artefact curation and resampling. No public reference dataset with published expected values exists for that part of the processing, which is in any case specific to the device pairing used here. Assurance for the upstream chain rests instead on three elements: operator review of the intermediate traces for every recording, with all automated suggestions advisory rather than applied; the internal consistency check described in section 3.1, in which the corrected inter-device offset reduced band-average phase discrepancies to approximately 1–3°; and the propagation of an explicit quality flag and artefact coverage figure to every exported result, so that the influence of pre-processing decisions on any individual estimate can be traced.

No formal cross-implementation agreement study has been performed for the present R pipeline. A comparison against an independent published implementation on the same recordings is planned and will be reported separately.

# 9 Software and data availability

The analysis pipeline is an original implementation in R of the methods specified in the CARNet white papers. The transfer function analysis follows the algorithmic specification of the CARNet reference implementation, against which it was verified as described in section 8; the reference scripts themselves are not redistributed. The “clintools: Tools for Clinical Research” is available from the CARNet site.

The exact software version used to generate the reported results is recorded in every output file together with the full parameter set, and should be cited by version and archive identifier rather than by repository name alone.

# 10 Reporting checklist

The following items, required or recommended by CARNet 2022 for dCA reporting, are addressed as indicated.

| **Item** | **Requirement** | **Location** |
| --- | --- | --- |
| Recording duration and conditions | REC #3 | Section 2 |
| Raw sampling frequency and anti-aliasing | REC #4 | Section 2, Table S1 |
| Beat-to-beat derivation method | REC #5 | Section 3.3 |
| Handling of artefacts and missing data | REC #6 | Sections 3.4, 3.5 |
| Resampling frequency and interpolation | REC #7 | Section 3.7 |
| Detrending, normalisation, filtering | REC #8, #9 | Table S3 |
| Window type, length, overlap, smoothing | REC #10–13 | Table S3 |
| Coherence threshold and its derivation | REC #14 | Section 4.1, Table S4 |
| Units of gain and phase | REC #15 | Section 4.4 |
| Spectral plots with SD curves | REC #15, #17 | Section 4.5 |
| MAP, mean CBFV, EtCO₂, HRV, spectral power | REC #17 | Section 6 |
| Step response plausibility | REC #18 | Section 7 |
| Time-domain correlation index | CARNet 2024 (4) | Section 5 |
| Exclusion criteria and participant flow | REC #14 | Section 7; main manuscript |

Supplementary Table S8. Reporting checklist against the CARNet 2022 recommendations.

# References

1. Claassen JAHR, Meel-van den Abeelen ASS, Simpson DM, Panerai RB; international Cerebral Autoregulation Research Network (CARNet). Transfer function analysis of dynamic cerebral autoregulation: a white paper from the International Cerebral Autoregulation Research Network. J Cereb Blood Flow Metab. (2016) 36:665–80.

2. Panerai RB, Brassard P, Burma JS, Castro P, Claassen JAHR, van Lieshout JJ, et al. Transfer function analysis of dynamic cerebral autoregulation: a CARNet white paper 2022 update. J Cereb Blood Flow Metab. (2023) 43:3–25.

3. Simpson DM, Payne SJ, Panerai RB. The INfoMATAS project: methods for assessing cerebral autoregulation in stroke. J Cereb Blood Flow Metab. (2022) 42:1084–97.

4. Kostoglou K, Bello-Robles F, Brassard P, Chacon M, Claassen JAHR, Czosnyka M, et al. Time-domain methods for quantifying dynamic cerebral blood flow autoregulation: review and recommendations. A white paper from the Cerebrovascular Research Network (CARNet). J Cereb Blood Flow Metab. (2024) 44:1480–514.

5. Olsen MH, Riberholt CG, Berg RMG, Møller K. Myths and methodologies: assessment of dynamic cerebral autoregulation by the mean flow index. Exp Physiol. 2024;109(4):614–623.

6. Claassen JAHR, Thijssen DHJ, Panerai RB, Faraci FM. Regulation of cerebral blood flow in humans: physiology and clinical implications of autoregulation. Physiol Rev. (2021) 101:1487–559.

**Part B — Acquisition and signal-processing methodology for cerebral vasomotor reactivity (VMR) testing**

| **Item** | **Value** |
| --- | --- |
| Analysis software | vmr-react — original R implementation for breath-holding and hyperventilation vasomotor reactivity testing |
| Software version | v0.3 (processing specification v0.3) |
| Runtime | R 4.3.3 |
| Corresponding author | Matteo Paolucci, IRCCS Istituto delle Scienze Neurologiche di Bologna / AUSL Bologna, Italy |

# 1 Purpose and scope

Cerebral vasomotor reactivity (VMR) quantifies the capacity of the cerebral arterioles to change calibre in response to a vasoactive stimulus, conventionally a change in arterial CO₂ tension. It is a distinct construct from dynamic cerebral autoregulation, which describes the response of cerebral blood flow to changes in perfusion pressure, and the two are assessed and reported separately in this study.

Unlike transfer function analysis, VMR testing has no equivalent of the CARNet white papers: there is no consensus specification of the manoeuvre, the baseline definition, the response metric, or the handling of concurrent haemodynamic change. Published breath-holding index values are consequently difficult to compare across studies. Part B therefore documents every analytical decision explicitly, including several for which the literature offers more than one defensible option, and states which option was adopted and why.

# 2 Test protocol

| **Parameter** | **Value** |
| --- | --- |
| Breath-holding (BH) test | Voluntary apnoea of approximately 30 s, starting after normal inspiration |
| Hyperventilation (HV) test | Approximately 30 s of paced hyperventilation at 60 breaths·min⁻¹, guided by an audible metronome |
| Interval between tests | 5 minutes |
| Position | Supine, at rest, quiet environment |
| Baseline | The 5 s immediately preceding the manoeuvre onset marker (see section 4.1) |
| Monitored channels | Bilateral middle cerebral artery CBFV; ECG; capnogram (end-tidal CO₂); continuous non-invasive arterial blood pressure |

Supplementary Table S9. Vasomotor reactivity test protocol.

**Rationale for concurrent ABP and EtCO₂ recording.** The breath-holding index is conventionally computed from CBFV alone. This is a substantive limitation: apnoea and hyperventilation both produce systemic haemodynamic changes, and a rise in CBFV during breath-holding may reflect a rise in arterial pressure rather than, or in addition to, CO₂-mediated vasodilation. Recording pressure and end-tidal CO₂ throughout the manoeuvre, and reporting the contribution of each, is a deliberate departure from routine practice and is described in section 5.

# 3 Acquisition and import

Acquisition uses the same equipment and settings as the autoregulation recordings described in the previous section (Viasonix Dolphin 4D with robotic 2 MHz probes for bilateral middle cerebral artery insonation; Finapres NOVA for continuous arterial pressure). Recordings are identified as breath-holding or hyperventilation by a token in the exported file name.

Files are exported by the Doppler system in a UTF-16 encoded, semicolon-separated text format with a variable-length instrument preamble. Import decodes the file through a connection rather than converting it in memory, and limits the header scan to the preamble, both to avoid the character-encoding corruption that occurs when the file is opened in spreadsheet software under a non-English locale and to keep import time acceptable on long recordings.

# 4 Signal processing

## 4.1 Manoeuvre markers

The start and end of each manoeuvre are defined by operator-confirmed markers. The operator inspects the capnogram to verify that the manoeuvre was actually performed as instructed — that apnoea was maintained rather than interrupted, and that hyperventilation achieved and sustained hypocapnia — and adjusts the markers accordingly.

An automated marker suggestion derived from the capnogram is available and is used to confirm the test duration: for breath-holding, the bounds of the flat capnogram segment; for hyperventilation, the hypocapnic window. As in the autoregulation pipeline, the suggestion is advisory only and is never applied without operator confirmation. Both the analysed duration and the original recording duration are reported for every test, so that a truncated or extended analysis window is visible in the output rather than concealed.

## 4.2 Beat detection

Beat detection follows the approach described in the previous section (dCA, 3.2), using the ECG R-wave as the primary fiducial with hybrid gap-filling from CBFV systolic peaks where the ECG drops out.

## 4.3 Mean flow velocity

Mean flow velocity (MFV) is computed as the time-averaged integral of the velocity envelope over each cardiac cycle — the quantity displayed live by the Doppler system as TAMAX/TAMEAN, and the same definition used for the autoregulation analyses.

**Deviation from common practice.** Many published vasomotor reactivity studies use the Gosling approximation, MFV = (PSV + 2·EDV)/3. That formula assumes a fixed waveform shape and is therefore not robust to changes in heart rate — precisely the condition induced by both breath-holding and hyperventilation. Using it would confound the reactivity estimate with the heart-rate response to the manoeuvre. The time-averaged integral is used throughout, and studies wishing to compare their values with ours should note which definition they employ.

## 4.4 End-tidal CO₂ extraction

End-tidal CO₂ is extracted per breath by segmenting expiratory runs above a detection threshold and taking the plateau maximum within each segment. Extracting the first threshold crossing instead latches onto the expiratory upstroke rather than the end-tidal plateau and systematically under-reads EtCO₂ by approximately a factor of two; the plateau-based extraction is therefore not a refinement but a correctness requirement.

Recordings in which the capnogram is absent or unusable are analysed without it and are flagged as such; no default or substituted CO₂ value is used. Indices computed without concurrent capnography carry a correspondingly weaker interpretation, since the stimulus magnitude is then unknown.

## 4.5 Baseline and outlier handling

Baseline MFV, ABP and EtCO₂ are computed over the 5 s immediately preceding the manoeuvre onset marker. Individual beats identified as artefactual are excluded through the same operator-confirmed curation interface used in the autoregulation pipeline, and either CBFV channel may be excluded in its entirety; an excluded side is exported as missing rather than as zero, with an EXCLUDE quality flag.

# 5 Reactivity indices

All indices are computed independently for the left and right middle cerebral artery. The percentage change in mean flow velocity is defined as

*ΔMFV% = 100 × (MFV_response − MFV_baseline) / MFV_baseline*

where MFV_baseline is the 5 s pre-manoeuvre mean and MFV_response is the extremum of the response, as defined below.

## 5.1 Definition of the response point

The response is taken as the greatest absolute change from baseline — the maximum for breath-holding, the minimum for hyperventilation — searched from manoeuvre onset and continuing into the recovery period rather than stopping at the end marker (7). The value at the nominal end of the manoeuvre is not used.

**Rationale.** The cerebrovascular response to apnoea is not synchronous with the manoeuvre: peak velocity commonly occurs shortly after breath release, and the CO₂ stimulus itself continues to rise into the early recovery. Reading the value at a fixed 30 s therefore systematically underestimates reactivity, and does so preferentially in patients with slow responses — the group of greatest clinical interest.

## 5.2 Index definitions

| **Index** | **Definition** | **Source** |
| --- | --- | --- |
| BHI / HVI (standard) | ΔMFV% divided by the manoeuvre duration in seconds | Markus and Harrison (8) |
| BHI to-max / HVI to-min | ΔMFV% divided by the latency in seconds from manoeuvre onset to the response extremum | Adapted from Nowakowska et al. (10) |
| BHAI / HVAI (acceleration index) | Slope of the most linear ascending (BH) or descending (HV) segment of the beat-to-beat MFV series, located by sliding-window ordinary least squares maximising R² over a minimum segment length; reported in cm·s⁻² and, normalised to baseline, in %·s⁻¹ | Alwatban et al. (14) |
| CVCi forms of all of the above | Each index recomputed on the cerebrovascular conductance series rather than on raw velocity (section 5.3) | This implementation |

Supplementary Table S10. Vasomotor reactivity index definitions. All indices are computed independently for each side, on both the raw velocity series and the conductance series.

**Note on the latency-normalised indices.** The numerator of the to-max and to-min indices is the same ΔMFV% used in the standard index; only the denominator differs, being the latency to the extremum rather than the manoeuvre duration. A literal steepest-gradient implementation — normalising by the interval over which the response rises most rapidly — was implemented and rejected: on weak responses the gradient estimate is dominated by beat-to-beat noise and the resulting index is unstable. The latency-based denominator retains the intended sensitivity to response speed while remaining well conditioned.

**Reporting both denominators.** Because the duration-normalised and latency-normalised forms answer different questions — response magnitude per unit of stimulus versus response speed — both are reported for every test, and the denominator used is stated with every value. Standard indices are additionally reported against the original recorded manoeuvre duration as well as the analysed duration, so that marker adjustment is transparent.

## 5.3 Separating the pressure and conductance contributions

The velocity response to a vasoactive manoeuvre reflects both the CO₂-mediated change in arteriolar calibre and any concurrent change in perfusion pressure. In some individuals the pressure contribution equals or exceeds the CO₂ contribution, in which case an apparently preserved breath-holding index substantially misrepresents vasomotor reactivity. Because the conventional index is computed from velocity alone, this confounding is invisible in routine reporting. The analysis therefore also computes a beat-by-beat index of cerebrovascular conductance:

*CVCi = MFV / MAP	(cm·s⁻¹·mmHg⁻¹)*

Every index in Supplementary Table S10 is recomputed on the CVCi series in addition to the raw velocity series, so that each test yields a velocity-based and a pressure-corrected value.

**Decomposition.** Since MFV = CVCi × MAP by construction, the following identity holds exactly in logarithms:

*Δln(MFV) = Δln(MAP) + Δln(CVCi)*

and therefore, to first order, ΔMFV% ≈ ΔMAP% + ΔCVCi% with a small interaction term. The following quantities are reported for every test and every side:

- bp_component = ΔMAP% — the pressure, or passive-perfusion, term;
- cond_component = ΔCVCi% — the conductance, or vasodilatory, term;
- bp_fraction = ΔMAP% / ΔMFV% and cond_fraction = ΔCVCi% / ΔMFV%;
- the exact logarithmic equivalents of each, retained for audit;
- the change in end-tidal CO₂ over the same interval, quantifying the stimulus actually delivered.

Systolic, mean and diastolic pressure and EtCO₂ extrema are located independently within the post-manoeuvre window, each with its own latency relative to the end marker, rather than being sampled at the instant of the velocity extremum. The response of each signal has its own time course, and forcing a common sampling instant would misattribute a lag to a difference in magnitude.

**Interpretation and its limit.** The conductance term is *not* a pure CO₂ term. It conflates CO₂-mediated vasodilation with active pressure autoregulation, which moves conductance in the opposite direction in order to defend flow. The choice between a conductance and a resistance formulation is itself not neutral: the two can support opposite conclusions about the magnitude of a vasomotor response when baseline flow differs substantially, which is the situation after unilateral large-vessel occlusion (11). More generally, the influence of mean arterial pressure on cerebrovascular reactivity estimates is a recognised methodological consideration rather than a solved problem (12), and absolute and relative expressions of a conductance response can diverge for the same data (13). Normalising by pressure therefore minimises but does not eliminate the influence of blood pressure on the reactivity estimate. The decomposition is accordingly presented as a descriptive attribution at the level of the individual test, and this caveat is printed on the report itself rather than left to the reader. The summary flags **BP-dom** and **CO2-dom** derive directly from which component predominates.

**Cohort-level partition.** At group level the pre-specified analysis is a general linear model of ΔMFV on ΔMAP, ΔEtCO₂ and their interaction, which estimates the two contributions across participants rather than within a single test (7). The within-test decomposition and the cohort model answer different questions, and both are reported.

# 6 Quality control

Every test carries explicit quality flags, exported with the numerical results.

| **Flag** | **Meaning** |
| --- | --- |
| bp_swing>10% | ΔMAP exceeded 10% between baseline and response point; the velocity-based index should not be interpreted without the conductance-based index alongside it (7) |
| BP-dom / CO2-dom | Indicates which component predominantly accounts for the observed velocity change (section 5.3) |
| EXCLUDE | The side was excluded at review; values exported as missing |
| capnogram absent | No usable capnogram; the delivered stimulus magnitude is unknown and indices are interpreted with reduced confidence (section 4.4) |
| marker adjusted | Analysed manoeuvre duration differs from the recorded duration; both are reported |

Supplementary Table S11. Vasomotor reactivity quality control flags.

Additional descriptive output supporting quality assessment includes peak systolic, end-diastolic and mean velocity together with the pulsatility index, reported at baseline and at the response point for both sides, and an ensemble-averaged beat waveform aligned to the ECG R-wave, overlaid for the two sides at baseline and at the response point. Waveform morphology changes that are inconsistent with a genuine vasomotor response — for example an insonation-angle shift during the manoeuvre — are identifiable from these panels.

# 7 Known limitations

- Breath-holding requires patient cooperation, which restricts its applicability in the acute stroke setting and introduces a selection effect that should be reported in the participant flow.
- The delivered CO₂ stimulus is not standardised across participants; capnography quantifies but does not equalise it. The achieved ΔEtCO₂ is reported alongside every index so that the stimulus magnitude is available to the reader, but the indices themselves are not normalised to it, and comparability with studies using controlled CO₂ administration remains limited.
- Transcranial Doppler measures velocity, not flow. All indices assume a constant insonated vessel diameter during the manoeuvre; this assumption is imperfect for the middle cerebral artery under a hypercapnic stimulus and is a recognised limitation of the technique rather than of this implementation.
- The absence of a consensus reporting standard for vasomotor reactivity means that absolute index values are not directly comparable across studies using different baselines, response definitions or velocity formulae. This is the reason for the level of detail in this document.
- No independent implementation exists against which this pipeline could be verified numerically, as is possible for transfer function analysis (Part A, section 8). Assurance rests on operator review of every trace, on advisory-only automation, and on the reporting of intermediate quantities — baseline, response point, latency, ΔEtCO₂ and ΔMAP — alongside every index, so that each value can be reconstructed and checked from the output itself.

# 8 Software and data availability

The vasomotor reactivity pipeline is an original R implementation. The availability arrangements are those set out in dCA section 9, and the same version and archive identifier conventions apply.

# References

7. Webb AJS, Paolucci M, Mazzucco S, Li L, Rothwell PM, Cohort OVSP. Confounding of Cerebral Blood Flow Velocity by Blood Pressure During Breath Holding or Hyperventilation in Transient Ischemic Attack or Stroke. *Stroke*. 2020;51(2):468-474. doi:10.1161/strokeaha.119.027829.

8. Markus HS, Harrison MJG. Estimation of cerebrovascular reactivity using transcranial Doppler, including the use of breath-holding as the vasodilatory stimulus. Stroke. (1992) 23:668–73.

9. Ringelstein EB, Sievers C, Ecker S, Schneider PA, Otis SM. Noninvasive assessment of CO₂-induced cerebral vasomotor response in normal individuals and patients with internal carotid artery occlusions. Stroke. (1988) 19:963–9.

10. Nowakowska-Kotas M, Lisiak W, Wiśniewska E, Budrewicz S, Wiśniewski P. Breath–holding index – a new approach to an old method. *Eur J Transl Clin Med*. 2025;8(1):10-18. doi:10.31373/ejtcm/204417.

11. O’Leary DS. Regional vascular resistance vs. conductance: which index for baroreflex responses? Am J Physiol. (1991) 260:H632–7. doi: 10.1152/ajpheart.1991.260.2.H632

12. Carr JMJR, Caldwell HG, Ainslie PN. Cerebral blood flow, cerebrovascular reactivity and their influence on ventilatory sensitivity. Exp Physiol. (2021) 106:1425–48. doi: 10.1113/EP089446

13. Keough JRG, Cates VC, Tymko MM, et al. Regional differences in cerebrovascular reactivity in response to acute isocapnic hypoxia in healthy humans: methodological considerations. Respir Physiol Neurobiol. (2021) 294:103770. doi: 10.1016/j.resp.2021.103770

14. Alwatban M, Truemper EJ, Al-rethaia A, Murman DL, Bashford GR. The Breath-Hold Acceleration Index: A New Method to Evaluate Cerebrovascular Reactivity using Transcranial Doppler. *J Neuroimaging*. 2018;28(4):429-435. doi:10.1111/jon.12508.
