# Supplementary material for: PFOCUS study protocol: a prospective case–control study investigating genetic, haemodynamic, and platelet function in migraine with aura with or without patent foramen ovale
Source: Front Neurol. 2026 Oct 16;17:1952744. doi: 10.3389/fneur.2026.1952744 (PMC13719609; doi:10.3389/fneur.2026.1952744)
Supplement: Supplementary file 2 [file Table_2.docx]

**Supplementary Material 2 — Statistical Analysis Plan (SAP)**

*Supplementary Material 2 to the PFOCUS study protocol*

**Study title:** Association between PFO and Migraine with Aura: a prospective, monocentric, observational case–control study with genetic and neurosonological analysis (PFOCUS).

**Protocol code:** PFOCUS (local ethics code CE25057). ClinicalTrials.gov: NCT07349004. Ethics reference: CE AVEC 293-2025-TESS-AUSLBO.

**Reference protocol version:** PFOCUS v1.0, 14 March 2025.

**SAP version:** 1.1 — September 2026 (revision of version 1.0 in response to peer review; see Section 19). **Status:** finalised before database lock / analysis of the primary endpoint.

**Prepared by:** M. Paolucci (Principal Investigator).

# **1. Introduction**

## **1.1 Purpose**

This Statistical Analysis Plan prospectively specifies the statistical methods that will be used to analyse the data of the PFOCUS study. It expands the statistical section of the protocol and defines the analysis populations, the derivation of endpoints, the handling of missing data, and the planned primary, secondary, and exploratory analyses. Any deviation from this SAP will be documented and justified in the final study report and in resulting publications.

## **1.2 Background and objectives**

PFOCUS investigates whether patients with migraine with aura (MA) and a patent foramen ovale (PFO) constitute a biologically distinct phenotype, characterised by genetic susceptibility (particularly within the Notch signalling pathway), cerebrovascular dysfunction, and a prothrombotic tendency, relative to MA patients without PFO. The study integrates genetic (whole-exome sequencing, WES), neurosonological (transcranial Doppler, TCD), and platelet-function data.

## **1.3 Hypotheses**

The primary null hypothesis is that the frequency of NOTCH3 gene variants does not differ between RLS-positive (RLS+) and RLS-negative (RLS−) patients with MA. The primary alternative hypothesis is that RLS+ patients carry NOTCH3 variants more frequently than RLS− patients. Secondary and exploratory hypotheses are analogously framed for the other genetic, haemodynamic, and platelet-function parameters and are regarded as hypothesis-generating.

# **2. Study design overview**

PFOCUS is a prospective, monocentric, observational, non-pharmacological, no-profit case–control study conducted at the IRCCS Istituto delle Scienze Neurologiche di Bologna. Consecutive adult patients with MA (ICHD-3) are enrolled and classified according to the presence of a right-to-left shunt (RLS) on contrast-enhanced TCD, used as a surrogate marker of PFO, as cases (RLS-positive, RLS+) or controls (RLS-negative, RLS−); patients who previously underwent percutaneous PFO closure are classified as RLS+ on the basis of pre-closure documentation. All assessments are performed during a single study visit. There is no intervention, no randomisation, and no allocation; the group variable (RLS status) is an observed characteristic, not an assigned condition.

# **3. Objectives and endpoints**

## **3.1 Primary endpoint**

Difference between RLS+ and RLS− patients in the frequency of NOTCH3 gene variants, defined as pathogenic/likely pathogenic mutations and “hot” and “cold” variants of uncertain significance (VUS), classified according to the criteria of the American College of Medical Genetics and Genomics (ACMG). The components of the primary endpoint (pathogenic/likely pathogenic variants, hot VUS, cold VUS) are also reported separately.

## **3.2 Secondary endpoints**

- difference in the frequency of pathogenic/likely pathogenic NOTCH3 variants alone (key secondary analysis, introduced in SAP v1.1; Section 8.4);
- difference in the frequency of variants in the other genes of the panel;
- correlation between variant frequency and right-to-left shunt size (Spencer Logarithmic Scale) in the RLS+ group;
- difference in the count of cerebral microemboli;
- differences in dynamic cerebral autoregulation and vasomotor reactivity (VMR) parameters;
- differences in platelet function / prothrombotic profile.

## **3.3 Exploratory endpoints**

- association between aura characteristics (age at onset, visual vs. sensory/complex aura, duration, frequency) and haemodynamic parameters;
- differences in platelet activation across aura subtypes;
- correlation between specific genetic variants and aura phenotypes;
- differences in the distribution/burden of MRI white-matter lesions.

# **4. Sample size**

Assuming a two-sided α = 0.05, power = 80%, an anticipated frequency of carriers of specific NOTCH3 polymorphisms of 15% in RLS− patients (as in Menon et al., 2011) and a doubling of this frequency in RLS+ patients, the required sample size is 240 participants, equally divided into 120 RLS+ and 120 RLS−. Because Menon et al. evaluated specific polymorphisms, the 15% figure is a plausible anchor rather than a precise estimate of the composite, whole-exome-based endpoint. It is nonetheless consistent with population data: in a Chinese population-based cohort, 13.6% of participants carried at least one rare NOTCH3 variant defined with the same minor allele frequency threshold (<1%) (Liu et al., 2023). The sensitivity of the detectable effect to this assumption was therefore quantified by computing, for 120 participants per group, the minimum detectable effect over a range of baseline frequencies (Supplementary Table S12): across baseline frequencies from 10% to 30%, the detectable odds ratio ranges from approximately 2.8 to 2.1, whereas power to detect a doubling falls to 58% at a baseline frequency of 10%. RLS status is determined in all participants (in those without an adequate transtemporal window, by TCCD through the transforaminal window); attrition for the primary endpoint is therefore essentially limited to sequencing failure, and with a 5% failure rate (114 participants per group) power remains approximately 78%. The sample size also reflects the maximum accrual achievable at a single centre within the funded 36-month recruitment period. The 1:1 ratio is consistent with the expected prevalence of PFO in MA (≈50%). The sample size is powered for the primary genetic endpoint only; secondary and exploratory analyses are not formally powered and are interpreted as hypothesis-generating. Calculations use the normal approximation for the comparison of two independent proportions.

**Supplementary Table S12.** Sensitivity of the sample size to the assumed baseline frequency of NOTCH3 variant carriers. Minimum detectable effect with 80% power and two-sided α = 0.05; 114 participants per group corresponds to a 5% sequencing failure rate. OR, odds ratio.

| **Baseline frequency (RLS−)** | **Detectable frequency in RLS+ (n = 120/group)** | **Detectable OR (n = 120/group)** | **Detectable OR (n = 114/group)** | **Power to detect a doubling (n = 120/group)** |
| --- | --- | --- | --- | --- |
| 5% | 16.0% | 3.63 | 3.73 | 31% |
| 10% | 23.4% | 2.75 | 2.82 | 58% |
| 15% (planning assumption) | 30.0% | 2.43 | 2.48 | 80% |
| 20% | 36.2% | 2.27 | 2.31 | 93% |
| 25% | 42.0% | 2.17 | 2.21 | 98% |
| 30% | 47.5% | 2.11 | 2.15 | >99% |

# **5. Analysis populations**

Analyses are performed on a per-domain basis, using all enrolled participants with valid, quality-controlled data for the relevant domain (complete-case approach). The populations are defined as follows:

| **Population** | **Definition** | **Primary use** |
| --- | --- | --- |
| Enrolled set | All participants who signed informed consent and met eligibility criteria. | Disposition, baseline characteristics. |
| Genetic analysis set | Participants with a valid WES result passing sequencing quality control. | Primary endpoint; genetic secondary/complementary analyses. |
| Neurosonology set | Participants with an interpretable TCD study (shunt grading, microemboli, and/or autoregulation recording meeting QC). In participants without an adequate transtemporal window, RLS is assessed by TCCD through the transforaminal window and dCA/VMR are not assessed. | Microemboli, autoregulation, VMR analyses (each further restricted to the metric-specific QC-valid subset). |
| Platelet analysis set | Participants with valid Multiplate aggregometry. | Platelet-function analyses. |
| Treatment-naïve platelet subset | Platelet analysis set participants with no ongoing aspirin or P2Y12-inhibitor therapy and no NSAID intake (including aspirin taken as acute migraine treatment) in the 48 hours preceding sampling. | Primary (confounder-free) platelet comparison. |

The number of participants analysed will be reported for every endpoint, together with the reasons for exclusion from each analysis.

# **6. General analysis principles**

- Analyses will be performed in R (version to be reported).
- All statistical tests are two-sided. The primary endpoint (pre-specified in SAP v1.0) and the key secondary analysis (Section 8.4; introduced in SAP v1.1) are single comparisons tested at α = 0.05 without adjustment; multiplicity for the other analyses is handled as specified in Section 6.1. Effect sizes with 95% confidence intervals (CIs) are emphasised over significance testing.
- Continuous variables are summarised as mean ± standard deviation or median (interquartile range), as appropriate to their distribution; categorical variables as counts and percentages. Normality is assessed graphically and, where needed, with the Shapiro–Wilk test.
- Between-group comparisons use the Student t-test or Mann–Whitney U test for continuous variables, and the chi-square test or Fisher's exact test for categorical variables, according to distribution and expected cell counts.
- Where a parameter is measured bilaterally (e.g., middle cerebral artery velocities), the mean of the valid sides is used; a side failing QC is excluded and the contralateral side retained.

## 6.1 Multiplicity

For secondary and complementary analyses, the false discovery rate (FDR) is controlled with the Benjamini–Hochberg procedure at q = 0.05 (Benjamini and Hochberg, 1995) within four pre-defined families of tests: (i) gene-level comparisons across the panel genes other than NOTCH3 (Section 9.1); (ii) SNP-level association tests (Section 10); (iii) haemodynamic parameters — microemboli count, TFA and Mxa indices, BHI and HVI (Sections 9.3, 9.4 and 11); and (iv) platelet-function parameters (Sections 9.5 and 12). Both unadjusted p-values and FDR-adjusted q-values are reported. Exploratory, subgroup and sensitivity analyses (Sections 14 and 15) are not adjusted; they are reported with effect sizes, 95% CIs and the number of tests performed, and are interpreted as hypothesis-generating.

# **7. Participant disposition and baseline characteristics**

A flow diagram will report the number of patients screened, eligible, enrolled, and included in each analysis population, with reasons for non-inclusion. Baseline demographic and clinical characteristics (age, sex, age at MA onset, aura type and duration, concomitant migraine without aura, current and lifetime peak attack frequency, chronic vs. episodic status, prophylactic and acute therapy, days since the last attack, interval from the last acute medication intake, vascular risk factors, family history) will be summarised overall and by group (RLS+ vs. RLS−) and compared descriptively. The complete list of clinical variables collected in the case report form is given in Supplementary Table S13.

**Supplementary Table S13.** Clinical variables collected in the case report form. (†) Field added in September 2026, in response to peer review, and collected prospectively from that date.

| **Domain** | **Variables** |
| --- | --- |
| Demographics | Age; sex; date of enrolment. |
| Migraine history | Age at MA onset; triggers (flickering or bright light, weather/season, noise/odours, psychological stress, physical exertion, menstrual cycle, changes in sleep or eating habits); aura type (visual, sensory, aphasic, motor); mean aura duration; side-locked aura; attacks without aura; tension-type headache; family history of migraine. |
| Disease activity | Number of MA attacks in the preceding 3 months; lifetime peak monthly MA frequency; current chronic migraine; past chronic migraine; days since the last migraine attack, with or without aura (†). |
| Acute treatment | Current acute treatments (paracetamol, NSAIDs, triptans, ditans, gepants, none); NSAID or other acute medication intake in the preceding 48 hours; time of the last acute medication intake. |
| Preventive treatment | For each class — lamotrigine, other antiepileptics, tricyclic antidepressants, beta-blockers, calcium-channel blockers, SSRIs/SNRIs, sartans, anti-CGRP(R) monoclonal antibodies, botulinum toxin, gepants, aspirin — status (never, previous, current) and response (effective and tolerated; ineffective or not tolerated). |
| Family history | Stroke/TIA; cerebral haemorrhage; dementia; PFO; migraine (with details). |
| Medical history and vascular risk factors | Hypertension; diabetes mellitus; atrial fibrillation; heart disease; dyslipidaemia; previous venous thrombosis; thrombophilia; ongoing aspirin, clopidogrel and oestrogen–progestogen therapy; active smoking or cessation <2 years; previous ischaemic stroke; previous cerebral haemorrhage; previous percutaneous PFO closure. |
| Brain MRI | Date of the most recent MRI; field strength and available sequences (†); Fazekas grade, deep and periventricular (0–3); lesions in the anterior temporal poles, external capsules and cerebellum; microbleeds or superficial siderosis; previous ischaemic or haemorrhagic lesions. |
| Post-test follow-up | MA attack during the one-hour observation after the contrast study. |

# **8. Primary analysis**

## **8.1 Variant classification**

Variants identified by WES (xGen Exome Research Panel v2, IDT; NovaSeq 6000, Illumina; hg38; BWA v0.7.17; GATK v4.2.0.0 multi-sample calling; annotation with Ensembl VEP v109.3, dbNSFP v4.3a and dbscSNV v1.1) within the NOTCH3 gene are classified per ACMG/AMP criteria as pathogenic, likely pathogenic, VUS, likely benign, or benign. The complete variant call set is analysed, irrespective of the filters applied in clinical reporting. Qualifying variants for the primary endpoint are rare (gnomAD r4.0.0 non-Finnish European minor allele frequency <1%) pathogenic/likely pathogenic mutations and VUS that are protein-altering or affect canonical splice sites; common polymorphisms are addressed in the complementary SNP-level analysis (Section 10). For the purposes of the registered primary endpoint, VUS are further operationally subclassified as hot or cold according to the laboratory classification used in the original protocol; these correspond, respectively, to the “VUS – leaning pathogenic” and “VUS – leaning benign” categories generated by the Franklin interpretation framework (Genoox, now QIAGEN; https://franklin.genoox.com). This subclassification is not an ACMG category and is used only to define the registered endpoint reproducibly. The same definition applies to all panel genes. Interpretation is performed blinded to RLS status and relies on gnomAD (Karczewski et al., 2020), ClinVar (Landrum et al., 2018), PanelApp, the published literature and the VarSome and Franklin platforms. Pathogenic and likely pathogenic variants are confirmed by Sanger sequencing. All software and database versions, including the Franklin version and date of access, and the transcripts used will be documented and date-stamped; for the primary analysis, the same pre-defined classification procedure is applied to all participants using a single database and software release (data freeze at the time of analysis), so that classification does not depend on the date of sequencing. Any subsequent reclassification, and its effect on the primary endpoint, will be reported separately.

## **8.2 Derivation and comparison**

For each participant, NOTCH3 variant carriage is derived as (i) a binary indicator of carrying at least one qualifying variant and (ii) the count of qualifying variants, overall and by category (pathogenic/likely pathogenic; hot VUS; cold VUS). The primary comparison between RLS+ and RLS− is the proportion of carriers of ≥1 qualifying NOTCH3 variant, tested with Fisher's exact test (chi-square if expected counts permit). Variant counts per participant are compared as a secondary summary using the Mann–Whitney U test (or a negative binomial model if overdispersion is present).

## **8.3 Effect estimates**

The primary effect measure is the odds ratio for NOTCH3 variant carriage (RLS+ vs. RLS−) with its 95% CI. Category-specific proportions (pathogenic/likely pathogenic, hot VUS, cold VUS) are reported with 95% CIs.

## 8.4 Key secondary analysis: pathogenic/likely pathogenic variants

The proportion of carriers of ≥1 pathogenic/likely pathogenic NOTCH3 variant is compared between RLS+ and RLS− with Fisher’s exact test and reported as an odds ratio with exact 95% CI, reported prominently alongside the primary endpoint, while clearly identified as a secondary analysis. This analysis was introduced in SAP v1.1 in response to peer review, before any analysis of study data; as a single comparison, it is tested at α = 0.05 without adjustment. Given the expected low frequency of such variants, this analysis is not formally powered.

# **9. Secondary analyses**

## **9.1 Other panel genes**

Variant frequency (carrier proportion and burden, defined as in Section 8) in the remaining panel genes is compared between groups using Fisher's exact test or the chi-square test. Results are reported with effect sizes and 95% CIs, and the false discovery rate is controlled across genes (family i, Section 6.1).

## **9.2 Variant frequency vs. shunt size**

Within the RLS+ group, the association between NOTCH3 (and panel) variant frequency and right-to-left shunt grade (Spencer Logarithmic Scale) is assessed with the Kruskal–Wallis test across shunt grades and with Spearman's rank correlation for the ordinal trend.

## **9.3 Cerebral microemboli**

The microemboli count (automated, Dolphin 4D) is compared between RLS+ and RLS− with the Mann–Whitney U test and summarised as median (IQR). FDR control is applied within family (iii) (Section 6.1).

## **9.4 Cerebral autoregulation and VMR**

See Section 11 for the detailed specification. Autoregulation and VMR parameters are compared between groups with parametric or non-parametric tests as appropriate. FDR control is applied within family (iii) (Section 6.1).

## **9.5 Platelet function**

See Section 12 for the detailed specification, including the handling of antiplatelet/NSAID therapy. FDR control is applied within family (iv) (Section 6.1).

# **10. Complementary SNP-level genetic analysis**

As a complementary analysis, common single nucleotide polymorphisms (SNPs) are coded by minor-allele count (0 = major-allele homozygote, 1 = heterozygote, 2 = minor-allele homozygote). Allele and genotype distributions are compared between RLS+ and RLS− using contingency tables with the chi-square test, under additive, dominant, and recessive models as appropriate. All SNPs are tested for Hardy–Weinberg equilibrium (in the control group), and linkage disequilibrium is evaluated with dedicated software. Candidate NOTCH3 polymorphisms previously associated with migraine (e.g., rs3815188, rs1043994) are examined specifically. FDR control is applied across SNP-level tests (family ii, Section 6.1).

# **11. Cerebral autoregulation and VMR — analysis specification**

Cerebral blood flow velocity (CBFV) in the middle cerebral arteries (TCD) and beat-to-beat arterial blood pressure (ABP, servo-controlled finger plethysmography / finometer) are co-recorded continuously for approximately 10 minutes at rest. A stable, artefact-free segment of at least 5 minutes is extracted for analysis; recordings or sides not meeting quality-control (QC) criteria (e.g., insufficient signal quality, inadequate ABP variability) are graded LOW_QUALITY and excluded, with the number of exclusions reported.

Dynamic cerebral autoregulation is quantified in two domains:

- **Frequency domain —** transfer function analysis (TFA) of the ABP–CBFV relationship, following the CARNet 2022 white paper, yielding coherence, gain (absolute and normalised), and phase in the very-low-frequency (0.02–0.07 Hz), low-frequency (LF, 0.07–0.2 Hz), and high-frequency (0.2–0.5 Hz) bands. LF phase and LF gain are the principal autoregulation metrics; parameters are estimated only where coherence exceeds the CARNet-recommended threshold.
- **Time domain —** the moving correlation coefficient Mx, specifically Mxa (correlation between spontaneous fluctuations of mean ABP and mean CBFV), computed per the CARNet 2024 recommendations. Higher (more positive) values indicate less efficient autoregulation.

Vasomotor reactivity (VMR) is assessed by the breath-holding index (BHI) and the hyperventilation index (HVI). Because hyperventilation frequently produces only a small change in end-tidal CO₂ and may fail QC, BHI is the primary VMR metric and HVI is secondary; standardised versions (BHI, HVI) are used. VMR and autoregulation parameters are compared between RLS+ and RLS− (Student t or Mann–Whitney U) and, exploratorily, correlated with shunt grade and aura characteristics. Sensitivity analyses adjust for, or stratify by, current vasoactive preventive treatment (calcium-channel blockers, beta-blockers) and consider the interval from the last migraine attack (Section 14; Supplementary Table S14).

# **12. Platelet function — analysis specification**

Platelet aggregation is measured by whole-blood impedance aggregometry (Multiplate®) as the area under the curve for the ADP, ASPI, and TRAP tests, together with the ADP/TRAP and ASPI/TRAP ratios. Because aspirin, P2Y12 inhibitors, and NSAIDs alter aggregometry, the primary (confounder-free) between-group comparison is performed in the treatment-naïve platelet subset (Section 5). A sensitivity analysis includes the full platelet analysis set, stratified by or adjusted for ongoing antiplatelet/NSAID therapy. Treatment-naïve status is defined as no ongoing aspirin or P2Y12-inhibitor therapy and no NSAID intake (including aspirin taken as acute migraine treatment) in the 48 hours preceding sampling; the recorded interval from the last acute medication intake is used in a further sensitivity analysis. Because antidepressants (SSRIs/SNRIs and tricyclics) may affect platelet function, sensitivity analyses also adjust for, or stratify by, current antidepressant use. Between-group comparisons use the Mann–Whitney U test; results are summarised as median (IQR).

**Supplementary Table S14.** Handling of concomitant treatments in the analyses.

| **Treatment** | **Outcomes potentially affected** | **Analytical handling** |
| --- | --- | --- |
| Ongoing aspirin or P2Y12 inhibitors | Platelet function | Excluded from the primary platelet comparison; included in a sensitivity analysis with adjustment or stratification. |
| NSAIDs (including aspirin as acute treatment) within 48 hours | Platelet function | As above; interval from the last acute medication intake examined in a further sensitivity analysis. |
| SSRIs/SNRIs, tricyclic antidepressants | Platelet function | Adjustment or stratification in sensitivity analyses. |
| Calcium-channel blockers, beta-blockers | dCA, VMR | Adjustment or stratification in sensitivity analyses. |
| Oestrogen–progestogen therapy | VMR, platelet function | Adjustment in sensitivity analyses. |
| Acute triptans, ditans, gepants | Cerebral haemodynamics (transient) | Interval from the last intake recorded and examined in sensitivity analyses. |

# **13. Missing data and data quality**

- A complete-case approach is used within each domain; no imputation is performed for the primary endpoint. The extent and pattern of missing data are described, and the number analysed is reported per endpoint.
- Reasons for missing or excluded measurements are recorded (e.g., haemolysed or clotted blood sample, test not performed, TCD/autoregulation QC failure, absent temporal window).
- Pre-specified range checks are applied to clinical fields; implausible or out-of-range values (e.g., attack-frequency fields containing evident data-entry artefacts) are reconciled against source documents and, if unresolved, set to missing. Robust summaries (median/IQR) are used for skewed clinical count variables.
- If missingness for the primary endpoint is non-trivial or appears non-random, a sensitivity analysis characterising analysed vs. non-analysed participants will be performed.

# **14. Subgroup and sensitivity analyses**

Subgroup analyses pre-specified in SAP v1.0, all exploratory and interpreted with caution: by sex (specified a priori, given the recognised female preponderance of both MA and PFO), by aura subtype (visual vs. sensory/complex), by age at aura onset (infant-juvenile vs adult), and by episodic vs. chronic migraine. Subgroup analysis added in SAP v1.1 (in response to peer review, before any analysis of study data): by attack frequency (<3 vs ≥3 attacks with aura in the preceding 3 months, i.e. approximately <1 vs ≥1 per month; attack frequency is also analysed as a continuous variable). Sensitivity analyses pre-specified in SAP v1.0: platelet analyses with vs. without treatment-naïve restriction (Section 12); autoregulation analyses with alternative QC thresholds; and, for the primary endpoint, alternative minor-allele-frequency thresholds for defining qualifying variants. Sensitivity analyses added in SAP v1.1: for the primary and key secondary endpoints, exclusion of participants with aura onset at 50 years or later; for haemodynamic and platelet analyses, exclusion of participants assessed within 72 hours of the last migraine attack (where this interval is available) and adjustment for treatment classes (Supplementary Table S14).

# **15. Exploratory analyses**

Exploratory analyses relate aura phenotype (age at onset, visual vs. sensory/complex aura, duration, frequency) to haemodynamic parameters (autoregulation, VMR) and to platelet activation, and examine MRI white-matter lesion burden. MRI is not a study procedure: the most recent clinically available scan is rated centrally by an expert neuroradiologist, blinded to RLS status and clinical data, if it meets the following minimum requirements (field strength ≥1.5 T; axial FLAIR or T2-weighted sequence with slice thickness ≤5 mm; T2*-weighted gradient-echo or susceptibility-weighted sequence for microbleeds and superficial siderosis, which are otherwise rated as not assessable). White-matter hyperintensities are graded with the Fazekas scale, separately for deep and periventricular white matter (Fazekas et al., 1987), and the other markers follow the STRIVE-2 definitions (Duering et al., 2023): lesions in the anterior temporal poles, external capsules and cerebellum, previous ischaemic or haemorrhagic lesions, and microbleeds or superficial siderosis. A random 20% sample of scans is independently rated by a second neuroradiologist, and inter-rater agreement is reported as weighted kappa for Fazekas grades and Cohen’s kappa for binary markers. Between-group comparisons use the Mann–Whitney U test for Fazekas grades and Fisher’s exact test for binary markers; volumetric quantification is not performed. Given scanner heterogeneity, variable timing, and incomplete availability, these analyses are strictly exploratory; the proportion of participants with usable MRI, scanner field strength, and time from the study visit will be reported.

# **16. Interim analyses and monitoring**

No formal interim analysis of the primary endpoint and no statistical stopping rules are planned. Recruitment and data-quality monitoring are conducted per Good Clinical Practice.

# **17. Deviations from the SAP**

Any deviation from this SAP, whether identified before or after unblinding of the analysis, will be documented with its rationale in the final study report and disclosed in resulting publications, distinguishing pre-specified from post-hoc analyses.

# **18. References**

**1.** Menon S, Cox HC, Kuwahata M, Quinlan S, MacMillan JC, Haupt LM, et al. Association of a Notch 3 gene polymorphism with migraine susceptibility. Cephalalgia. 2011;31(3):264–270.

**2.** Richards S, Aziz N, Bale S, Bick D, Das S, Gastier-Foster J, et al. Standards and guidelines for the interpretation of sequence variants: a joint consensus recommendation of the ACMG and the AMP. Genet Med. 2015;17(5):405–424.

**3.** Panerai RB, Brassard P, Burma JS, Castro P, Claassen JAHR, van Lieshout JJ, et al.; Cerebrovascular Research Network (CARNet). Transfer function analysis of dynamic cerebral autoregulation: a CARNet white paper 2022 update. J Cereb Blood Flow Metab. 2023;43(1):3–25.

**4.** Kostoglou K, Bello-Robles F, Brassard P, Chacon M, Claassen JAHR, Czosnyka M, et al. Time-domain methods for quantifying dynamic cerebral blood flow autoregulation: review and recommendations. A white paper from CARNet. J Cereb Blood Flow Metab. 2024;44(9):1480–1514.

**5.** Headache Classification Committee of the International Headache Society (IHS). The International Classification of Headache Disorders, 3rd edition. Cephalalgia. 2018;38(1):1–211.

**6.** Benjamini Y, Hochberg Y. Controlling the false discovery rate: a practical and powerful approach to multiple testing. J R Stat Soc Series B Stat Methodol. 1995;57(1):289–300.

**7.** Landrum MJ, Lee JM, Benson M, et al. ClinVar: improving access to variant interpretations and supporting evidence. Nucleic Acids Res. 2018;46(D1):D1062–D1067.

**8.** Karczewski KJ, Francioli LC, Tiao G, et al. The mutational constraint spectrum quantified from variation in 141,456 humans. Nature. 2020;581(7809):434–443.

**9.** Liu ZY, Zhai FF, Liu JY, Zhou YJ, Shu MJ, Huang XH, et al. Pattern of brain parenchymal damage related to cerebral small vessel disease in carriers of rare NOTCH3 variants. Neurology. 2023;101(20):e1979–e1991.

**10.** Fazekas F, Chawluk JB, Alavi A, Hurtig HI, Zimmerman RA. MR signal abnormalities at 1.5 T in Alzheimer’s dementia and normal aging. AJR Am J Roentgenol. 1987;149(2):351–356.

**11.** Duering M, Biessels GJ, Brodtmann A, et al. Neuroimaging standards for research into small vessel disease—advances since 2013. Lancet Neurol. 2023;22(7):602–618.

# **19. Revision history**

| **Version** | **Date** | **Author** | **Description** |
| --- | --- | --- | --- |
| 1.0 | 14 March 2025 | Matteo Paolucci | First version, finalised before analysis of the primary endpoint. |
| 1.1 | September 2026 | Matteo Paolucci | Revised in response to peer review, before any analysis of study data: RLS terminology; operational hot/cold VUS definition and classification data freeze and MAF threshold; key secondary endpoint (pathogenic/likely pathogenic variants); sample-size sensitivity analysis; multiplicity strategy (FDR); operational definition of treatment-naïve status and handling of treatments; additional subgroup and sensitivity analyses; MRI requirements and rating. |
